# Cost-effective whole-cell biosynthesis of ursodeoxycholic acid using engineered *Escherichia coli* with a multienzyme cascade

**DOI:** 10.3389/fmicb.2025.1538237

**Published:** 2025-01-22

**Authors:** Xue Zhang, Jiagang Xin, Mengyu Liu, Yue Zhang, Haoni Luan, Wei Feng, Fei Wang, Wei Xu, Peng Song

**Affiliations:** ^1^School of Pharmaceutical Sciences and Food Engineering, Liaocheng University, Liaocheng, China; ^2^National Key Laboratory of Macromolecular Drug Development and Manufacturing, Liaocheng, China; ^3^Shandong Aobo Biotech Co. Ltd., Liaocheng, China

**Keywords:** chenodeoxycholic acid, ursodeoxycholic acid, whole-cell catalysis, 7**α**-hydroxysteroid dehydrogenase, 7**β**-hydroxysteroid dehydrogenase, lactate dehydrogenase, glucose dehydrogenase

## Abstract

Ursodeoxycholic acid (UDCA) can be used as a drug to treat various liver and bile diseases. Currently, the biological synthesis of UDCA is predominantly conducted via a two-step enzymatic process in which synthesis is catalyzed by 7α-hydroxysteroid dehydrogenase (7α-HSDH) and 7β-hydroxysteroid dehydrogenase (7β-HSDH) in succession, utilizing chenodeoxycholic acid (CDCA) as the substrate. In this study, an engineered *Escherichia coli* (*E. coli*) strain, designated UCA23, was constructed. This strain coexpressed four enzymes under the control of three independent T7 promoters: lactate dehydrogenase (LDH) derived from *Lactobacillus delbrueckii*, glucose dehydrogenase (GDH) derived from *Priestia megaterium*, 7α-HSDH derived from *E. coli*, and 7β-HSDH derived from *Ruminococcus torques*, enabling the whole-cell catalytic synthesis of UDCA from CDCA. This study systematically optimized the reaction parameters, including temperature, pH, and the addition of organic solvents and surfactants, for the whole-cell catalytic synthesis of UDCA by UCA23, and at the 2 L level, a UDCA conversion rate of 99% was achieved with 100 mM CDCA in 2 h, which is the highest level of conversion of a high-concentration CDCA substrate reported to date.

## 1 Introduction

**Figure 1:**
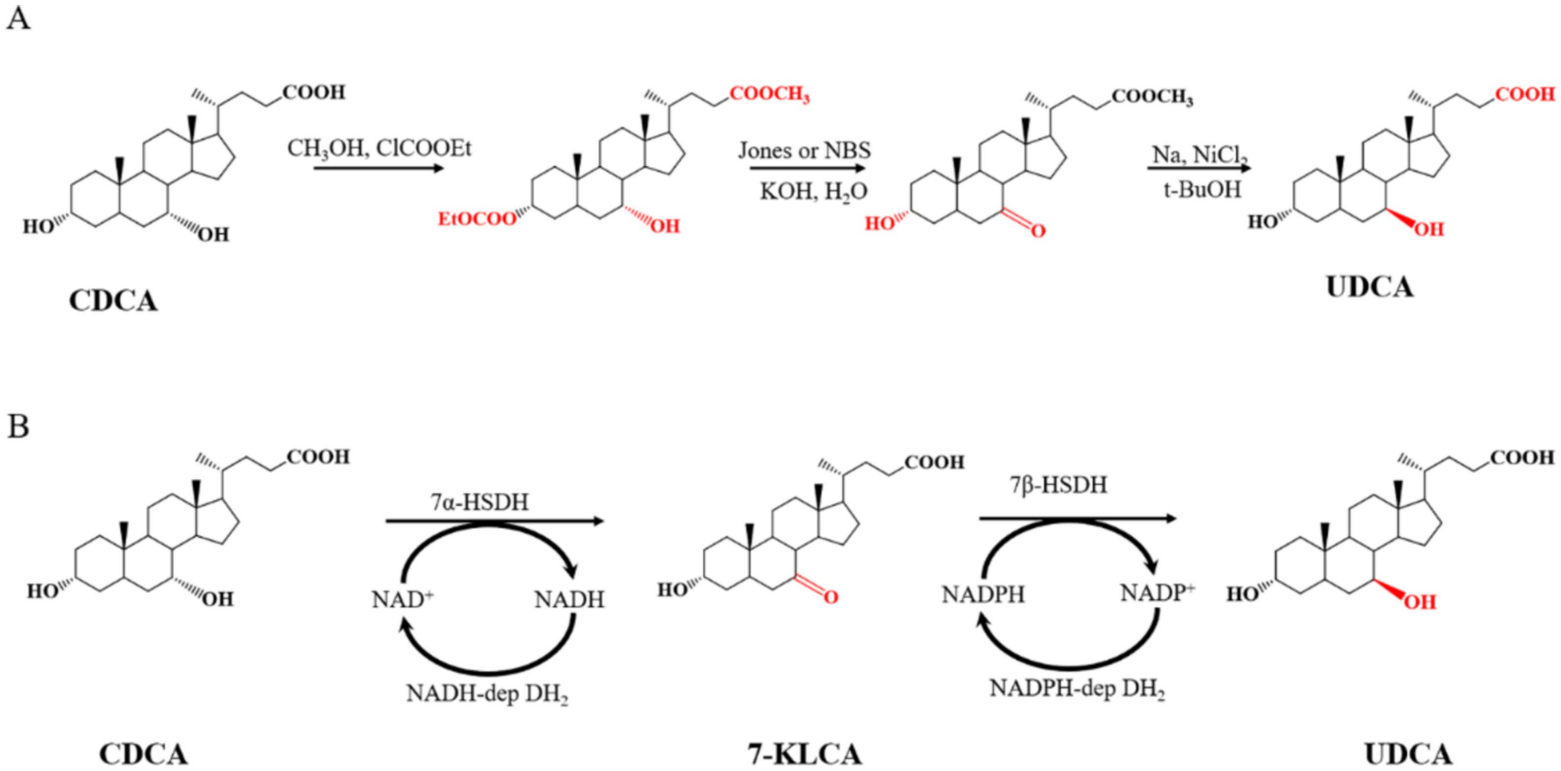
Synthesis of UDCA from CDCA by **(A)** chemical method and **(B)** enzyme-catalyzed method.

Ursodeoxycholic acid (UDCA) is used clinically to treat a variety of liver and bile diseases, such as gallstones, viral hepatitis, fatty liver and cirrhosis (Lindblad et al., 1998; Kim et al., 2018; Song et al., 2023; Cifuentes-Silva and Cabello-Verrugio, 2024; van Hooff et al., 2024; Wang et al., 2024). To date, UDCA is the only drug approved by the US Food and Drug Administration (FDA) for the treatment of primary biliary cirrhosis. The synthesis of UDCA from chenodeoxycholic acid (CDCA) is economical because chicken galls and duck galls, which are byproducts of poultry processing, are inexpensive and abundant (Yeh and Hwang, 2001). UDCA is chemically or enzymatically synthesized from CDCA (Tonin and Arends, 2018; Song et al., 2023). In traditional chemical synthesis, with the methylation of CDCA, the hydroxyl group at the C3 position is protected, and then Jones reagent or N-bromosuccinimide (NBS) is used to oxidize the hydroxyl group at the C7 position. Finally, UDCA is obtained from the reduction intermediate product in tert-butanol (t-BuOH) with sodium metal and nickel dichloride. The UCDA yield with this method is only 43% (Guo et al., 2020). The chemical synthesis route for UDCA is shown in Figure 1A.

The enzymatic synthesis of UDCA requires only two steps: the first step is the 7α-hydroxysteroid dehydrogenase (7α-HSDH)-mediated oxidation of CDCA to generate 7 K-LCA (7-keto-lithocholic acid), and the second step is the 7β-hydroxysteroid dehydrogenase (7β-HSDH)-mediated reduction of 7 K-LCA to generate UDCA. These two steps require the coenzymes NAD^+^ and NADPH, respectively. The regeneration and utilization of the expensive coenzymes NAD^+^ and NADPH, respectively, are achieved through NADH-dependent and NADPH-dependent dehydrogenases (Zheng et al., 2015; Ji et al., 2016; Zheng et al., 2017a; Zheng et al., 2017b; Tonin and Arends, 2018). The route of CDCA enzymatic synthesis of UDCA is shown in Figure 1B. Compared with chemical methods, enzymatic synthesis has become the mainstream method of industrial production because of its low degree of pollution and high efficiency.

The enzymatic synthesis of UDCA can be performed via a “one-pot one-step” or “one-pot two-step” method. In “one-pot one-step” (a two-step reaction is simultaneously conducted in one pot without intermediate treatment) synthesis, four enzymes (7α-HSDH, 7β-HSDH, NADH-dependent dehydrogenase and NADPH-dependent dehydrogenase), substrates, auxiliary ingredients (sodium pyruvate, glucose, NAD^+^, and NADP^+^) and buffer are added to the reaction system at the same time, and the conversion rate is generally less than 80% (Pedrini et al., 2006; Zheng et al., 2015; Yang et al., 2023). During the “one-pot two-step” (inactivating the enzymes at the end of the first step and proceeding to the second step of the reaction) synthesis, 7α-HSDH, NADH-dependent dehydrogenase, substrates, sodium pyruvate and NAD^+^ are added for the first step of the reaction to convert CDCA to 7 K-LCA. After the reaction is complete, the enzymes are deactivated at 80–90°C and then cooled. 7β-HSDH, NADPH-dependent dehydrogenase, glucose and NADP^+^ are added for the second step to complete the conversion from 7 K-LCA to UDCA, with a conversion rate of over 80% (Kole and Altosaar, 1985; Bovara et al., 1993; Zheng et al., 2015; Zheng et al., 2017b; Zhang et al., 2019). However, both methods have drawbacks. First, in the “one-pot one-step” reaction, because there is a reaction balance, the highest product conversion rate achievable is only approximately 80%. Therefore, the conversion rate of the “one-pot one-step” reaction is low, resulting in difficulty in separating products in the later stage. Second, in the “one-pot two-step” reaction, after the first step of the reaction, it is necessary to heat the mixture to more than 80°C to deactivate the enzyme and then cool the mixture for the second step of the reaction (Zheng et al., 2015; Zheng et al., 2017b). Although this method has a high conversion rate of UDCA, it is carried out with a low concentration of substrate and has a small-scale reaction system; it also increases energy consumption but reduces production efficiency. Third, the coenzyme consumption of the two methods is too high, and the need to add 0.2–0.5 mM coenzyme I and coenzyme II greatly increases the production cost (Zheng et al., 2015; Tonin and Arends, 2018; Wang et al., 2023; Yang et al., 2023).

**Figure 2:**
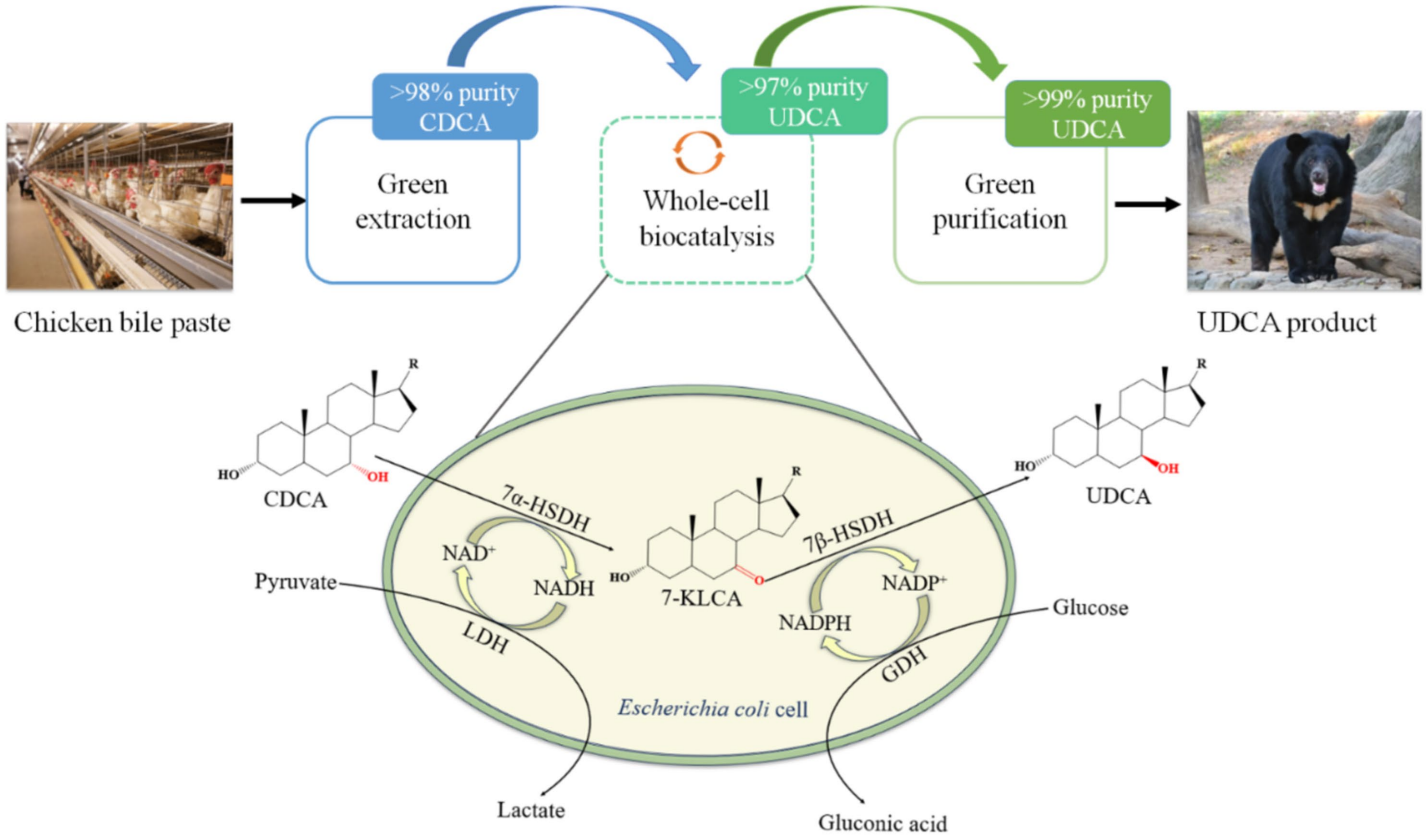
Schematic diagram of whole-cell catalytic synthesis of UDCA using CDCA as substrate.

Whole-cell catalysis is a new catalytic mode with many advantages, especially in multistep enzyme-catalyzed reactions, showing great application prospects and industrial value in the synthesis of many drug intermediates (Sun et al., 2013; Schrittwieser et al., 2018; Wu and Li, 2018). A schematic diagram of the whole-cell catalytic synthesis of UDCA from CDCA is shown in Figure 2. Compared with enzyme catalysis, whole-cell catalysis has two obvious advantages (Schmid et al., 2001; Fontanille et al., 2006; Carballeira et al., 2009; Sun et al., 2013; Park et al., 2020; Chen et al., 2021; Shin et al., 2021): first, lysis of fermentative bacteria is not needed, and instead, the cells are used directly as reactors after collection, reducing the number of processing steps and the impurities in the reaction system and facilitating the subsequent purification process; and second, the use of an intracellular coenzyme regeneration system (such as NAD^+^ or NADP^+^) to achieve *in situ* regeneration of coenzymes, with no need to artificially add coenzymes or reduce the amount of coenzymes added, thus reducing production costs (Rudroff, 2019; Mordhorst and Andexer, 2020; Wang et al., 2023).

The synthesis of UDCA by whole-cell catalysis using CDCA as a substrate starts with wild-type bacteria, as these bacteria naturally contain two enzymes, 7α-HSDH and 7β-HSDH, that can convert CDCA to UDCA (Hirano and Masuda, 1982; Macdonald and Hutchison, 1982; Macdonald et al., 1982; Sutherland and Macdonald, 1982; Sutherland et al., 1984; Medici et al., 2002). However, whole-cell catalysis via wild-type bacteria has the following limitations: wild-type bacteria are normally difficult to grow, especially if enzyme expression is related to anaerobic conditions; the pathogenicity of these bacteria poses a problem for their use in the pharmaceutical industry; and additional steps of purification and sterilization are necessary to obtain a safe product for the market (Tonin and Arends, 2018). Owing to the low substrate concentration and low conversion rate of wild-type bacteria (Medici et al., 2002; Lepercq et al., 2004), engineered strains have been subsequently constructed to achieve a relatively high conversion rate. Shi et al. constructed an engineered strain to coexpress 7α-HSDH and 7β-HSDH in *E. coli* to catalyze the conversion of taurochenodeoxycholic acid (TCDCA) to tauroursodeoxycholic acid (TUDCA). Under the optimal conditions, *E. coli* containing 7α-HSDH and 7β-HSDH were demonstrated to produce up to 1.61 ± 0.13 g/L TUDCA from 3.23 g/L TCDCA. TCDCA or TUDCA is a bound bile acid formed by condensing the carboxyl group of CDCA or UDCA with the amino group of taurine, and therefore, this method can also be used to convert CDCA to UDCA (Shi et al., 2017). Chen et al. constructed a coenzyme regeneration system consisting of flavin redox enzyme (FR)/flavin mononucleotide (FMN). In their concurrent one-pot preparation, the tolerated substrate concentration was low. At higher substrate concentrations, the complete epimerization of 7α-OH and enzyme activity were inhibited. When the substrate concentration was 40 mM, the conversion rate reached only 60%. Moreover, an additional 0.15 mM NAD^+^ and NADP^+^ was needed, and this system also required the costly addition of 0.22 mM FMN for effective NAD^+^ recycling (Chen et al., 2019). Both systems remain far from ready for industrial applications. Compared with previous reports, the present study revealed a reduced quantity of costly coenzymes and an increased substrate loading capacity and reaction system. This study paves the way for the industrial production of UDCA by whole-cell catalysis.

In this study, 7α-HSDH from *E. coli*, LDH from *Lactobacillus delbrueckii*, 7*β*-HSDH from *Ruminococcus torques*, and GDH derived from *Priestia megaterium*, whose functions have been identified in our laboratory, were simultaneously expressed in the same *E. coli* strain, and protein expression was optimized. The results of the whole-cell catalytic synthesis of UDCA from CDCA in this study revealed the highest spatiotemporal conversion and substrate loading capacity achieved to date and that this method is currently the least expensive method of UDCA synthesis.

## 2 Materials and methods

### 2.1 Chemicals and reagents

Unless otherwise stated, all other chemicals and reagents used in this work were obtained commercially and were of reagent grade. CDCA, 7 K-LCA, UDCA, isopropyl β-D-1-thiogalactopyranoside (IPTG), kanamycin, ampicillin and chloromycetin were purchased from Sigma–Aldrich Corp. (St. Louis, MO, United States). Ammonia was purchased from Yantai Far East Fine Chemical Co., Ltd. (Yantai, China). The reduced/oxidized forms of NADH/NAD^+^ and the reduced/oxidized forms of NADPH/NADP^+^ were purchased from Solarbio Co., Ltd. (Beijing, China). Methanol, ethanol, acetone, toluene, Triton X-100, Tween-20, nonyl phenoxypoly ethanol (NP-40) and polyoxyethylene lauryl ether (Brij-35) were purchased from Sangon Co., Ltd. (Shanghai, China).

The plasmids were extracted via the Plasmid Mini Kit or the DNA Purification Kit (Tiangen, Beijing, China). Pyrobest^™^ DNA polymerase (Takara, Dalian, China) was used for PCR. Restriction endonucleases and the GeneArt^™^ Gibson Assembly HiFi Cloning Kit were purchased from Thermo Scientific (Waltham, USA). The intracellular concentrations of NAD^+^/NADH and NADP^+^/NADPH were determined by using an NAD^+^/NADH Kit and an NADP^+^/NADPH Kit (Beyotime Biotechnology, Shanghai, China), respectively.

### 2.2 Bacterial strains, culture media and preparation of engineered *Escherichia coli*

*E. coli* BL21 (DE3) and recombinant *E. coli* BL21 (DE3) cells expressing heterologous proteins were cultivated in Luria–Bertani (LB) media (5 g/L yeast extract, 10 g/L tryptone, and 10 g/L NaCl). To maintain the plasmids in the engineered strains or provide selective pressure, kanamycin (30 μg/mL), ampicillin (100 μg/mL) or chloromycetin (34 μg/mL) was added to the medium.

The fermentation medium in a 5 L fermenter for producing recombinant *E. coli* contained the following components: 5 g/L glycerol, 15 g/L yeast extract, 20 g/L peptone, 4 g/L NaCl, 10 g/L K_2_HPO_4_, 2.5 g/L NaH_2_PO_4_·2H_2_O, 2.5 g/L (NH_4_)_2_SO_4_, 1.5 g/L MgSO_4_·7H_2_O, and 1 mL/L trace element solution. The trace element solutions were prepared as follows: 2.8 g/L FeSO_4_·7H_2_O, 2.8 g/L CoSO_4_·7H_2_O, 2 g/L MnCl_2_·4H_2_O, 1.5 g/L CaCl_2_·2H_2_O, 0.3 g/L ZnSO_4_·7H_2_O, 0.2 g/L CuCl_2_·2H_2_O, and 0.3 g/L MgSO_4_·7H_2_O were dissolved in water. The pH of the fermentation medium was adjusted to 7.0 with ammonia. When needed, 30 μg/mL kanamycin was added. The inoculated engineered strains were grown overnight at 37°C and 220 rpm in 50 mL of LB medium. These cultures were inoculated into a 5 L fermenter (Hub230-5 L, Holves, Anhui, China) containing 3 L of fermentation medium. The fermentation temperature was controlled at 37°C, and the pH was maintained at 7.0 by adding ammonia. Antifoam 204 (Shanghai Macklin Biochemical Technology Co., Ltd., Shanghai, China) was added for foam control during fermentation. The air flow rate was maintained at 1.3 VVM. During batch cultivation, the dissolved oxygen content was increased to 30% or more by increasing the stirring speed (200–800 rpm) and/or aeration. Expression was induced when the cell culture was at an OD_600_ of 10 by adding IPTG at a final concentration of 0.5 mM and adjusting the temperature to 30°C. The growth of the bacterial culture was determined by measuring the OD_600_ via an ultraviolet spectrophotometer (Shanghai Yoke Instrument and Meter Co., Ltd., Shanghai, China). When the enzyme activity peaked, the cells were collected for the next step, and enzyme activity was determined by the methods described in the following text.

### 2.3 Gene cloning and construction of recombinant plasmids

The genes encoding 7α-HSDH, LDH, 7β-HSDH and GDH were synthesized by GenScript (Nanjing, China) and subjected to codon optimization for expression in *E. coli*. All primers used to amplify the genes in this study are listed in Supplementary Table S1, and the recombinant vectors constructed are shown in Supplementary Table S2.

#### 2.3.1 Construction of single-enzyme expression plasmids

The LDH fragments were amplified by the primers LDH-F and LDH-R with a synthetic plasmid as a template and then inserted into the *E. coli* expression vector pET28a (+) via the *Nco* I and *Hind* III restriction enzymes to construct pET28a-ldh. pET28a-7α, pET28a-7β, and pET28a-gdh were constructed using the same method.

#### 2.3.2 Construction of dual-enzyme coexpression plasmids

The LDH/7α-HSDH gene combination and GDH/7β-HSDH gene combination were inserted into the *E. coli* expression vectors pETDuet-1, pRSFDuet-1 and pACYCDuet-1 (all three plasmids were for the coexpression of two target genes). The LDH and 7α-HSDH fragments were amplified with the primers LDH-F/LDH-R and 7α-F/7α-R and then inserted into multiple cloning site 1 (MCS1) digested with *Nco* I/*Hind* III and multiple cloning site 2 (MCS2) digested with *Nde* I/*Xho* I, respectively, to construct pETDuet-ldh-7α. Similarly, LDH and 7α-HSDH were inserted into pRSFDuet-1 and pACYCDuet-1 to construct pRSFDuet-ldh-7α and pACYCDuet-ldh-7α. The GDH/7β-HSDH gene combination was inserted in the same way as LDH/7α-HSDH to construct pACYCDuet-gdh-7β, pETDuet-gdh-7β, and pRSFDuet-gdh-7β. As a control, a pRSFDuet-7β-7α without coenzyme regeneration was also constructed.

#### 2.3.3 Construction of four-enzyme coexpression plasmids

The gdh-7β fragments were amplified by the primers GDH-F and 7β-R with pRSFDuet-gdh-7β as the template, and the ldh-7α fragments were amplified by the primers LDH-F and 7α-R with pRSFDuet-ldh-7α as the template. The gdh-7β and ldh-7α fragments were subsequently inserted into MCS1 digested with *Nco* I/*Hind* III and MCS2 digested with *Nde* I/*Xho* I, respectively, to construct pACYCDuet-gdh-7β-ldh-7α. The four-enzyme coexpression plasmids pETDuet-gdh-7β-ldh-7α and pRSFDuet-gdh-7β-ldh-7α were constructed with the same method as pACYCDuet-gdh-7β-ldh-7α. When promoters between genes needed to be removed, primers were designed separately, and overlapping PCR was performed to amplify the gb and la fragments and connect them to the pRSFDuet-1 vector via the same method as that was used for pACYCDuet-gdh-7β-ldh-7α to construct pRSFDuet-gb-la. Similarly, the gb/ldh-7α gene combination and gdh-7β/la gene combination were inserted by the same method to construct pRSFDuet-gb-ldh-7α and pRSFDuet-gdh-7β-la.

### 2.4 Enzyme expression and activity assays

The recombinant plasmids harbored in *E. coli* Top10 cells were extracted and then transformed into *E. coli* BL21 (DE3) cells for heterologous enzyme expression. The recombinant *E. coli* BL21 (DE3) cells (Supplementary Table S3) were cultivated in LB media supplemented with appropriate antibiotics. When the OD_600_ reached 0.4–0.6, 0.5 mM IPTG was added to the culture broth, followed by culture for an additional 4 h at 30°C. The recombinant cells were harvested by centrifugation at 8000 × *g* for 10 min at 4°C. A 1 g cell pellet was resuspended in 10 mL of phosphate buffer (75 mM, pH 8.0) and disrupted by sonication. The samples were sonicated for 100 × 5 s (operated 100 times with 5 s still time between each operations) and the acoustic power was controlled at 400 W. The supernatant was collected by centrifugation at 8,000 × g for 20 min at 4°C to remove the cell debris. The soluble protein sample was used to determine activity, and SDS–PAGE was performed to assess protein expression (12% separation gel).

The activities of the four dehydrogenases were assayed by spectrophotometrically measuring the reduction of NAD(P)^+^ or the oxidation of NAD(P)H at 340 nm and 25°C. For the determination of 7α-HSDH activity, the following enzyme mixture in a total volume of 2 mL was used: 80 mM CDCA in 75 mM phosphate buffer solution (PBS) (pH 8.0) and 5 mg/mL NAD^+^. LDH activity was determined in 2 mL of assay mixture containing 100 mM sodium pyruvate in 75 mM PBS (pH 8.0) and 5 mg/mL NADH. For the determination of 7β-HSDH activity, the following enzyme mixture in a total volume of 2 mL was used: 80 mM 7 K-LCA in 75 mM PBS (pH 8.0) and 5 mg/mL NADPH. GDH activity was determined in 2 mL of assay mixture containing 100 mM glucose in 75 mM PBS (pH 8.0) and 5 mg/mL NADP^+^. All the enzyme solutions used 2 μL in the above systems. One unit of activity is defined as the amount of enzyme that is needed to reduce/oxidize 1 μmol of NAD(P)^+^/NAD(P)H in 1 min under the assay conditions described above. Each measurement was performed at least three times.

## 3 Whole-cell biotransformation and optimization of conditions

### 3.1 Whole-cell catalytic analysis of different engineered *Escherichia coli* combinations

The cells of the single-enzyme-expressing strains, dual-enzyme-coexpressing strains and four-enzyme-coexpressing strains were collected by centrifugation at 8,000 × g for 10 min after the cells were induced for 4 h in shaking flasks, after which the enzyme activity of each strain was assessed. The amount of wet cells added to the reaction system was consistent to ensure that each corresponding enzyme activity remained consistent and to compare the catalysis in the three types of whole cells. The whole-cell catalytic synthesis of UDCA from CDCA was performed under the following conditions in a 50 mL reaction system: 100 mM CDCA, 75 mM PBS buffer (pH 8.0), 150 mM sodium pyruvate, 150 mM glucose, 7α-HSDH (10 U/mL), LDH (10 U/mL), 7β-HSDH (5 U/mL), GDH (5 U/mL), stirring at 150 rpm, 25°C, and reacting for 4 h. Notably, for the strains coexpressing different enzymes, the addition of wet cells was guided by the enzyme with the lowest activity, which met the requirements of the standard.

### 3.2 Optimization of the whole-cell catalytic reaction conditions

Several critical factors affecting the reaction were tested in a 50 mL system to optimize whole-cell biotransformation. The temperature was tested from 20°C to 40°C, the pH was tested from 6.0 to 9.5, the PBS concentration was tested from 25 to 150 mM, the effects of organic solvents and surfactants were tested in the reaction system via the addition of 5% organic solvents and 0.5% surfactants, and the effect of the NADP^+^ concentration ranging from 0 to 50 μM was tested. The reaction system contained 100 mM CDCA, 150 mM sodium pyruvate and 150 mM glucose. Scale-up reactions were performed at a 2 L scale using the best catalytic cells verified in Sections 3.1 and 3.2 under the optimal catalytic conditions. The wet cells used were obtained with a 5 L fermenter.

### 3.3 Whole-cell recovery and recycling

The same batch of cells was subjected to three consecutive reactions under the verified optimal conditions to investigate the reusability of the optimal whole-cell mixture. After the completion of the first-round reaction, the pH of the reaction mixture was adjusted to 9.0 with a 4 M NaOH solution to dissolve the UDCA product, the supernatant and the cells were separated by centrifugation at 8,000 × g and 25°C, and the collected cells were used for the second-round reaction. After the second-round reaction was complete, the same treatment was performed, and the third-round of the reaction was performed.

## 4 Analytical methods

High-performance liquid chromatography (HPLC) was used to analyze the levels of CDCA, 7 K-LCA and UDCA in the reactions. Before analysis, the reaction mixture was terminated by boiling for 5 min and further centrifuged at 8,000 × g for 15 min. Then, chromatography grade methanol (1 mL) was added to the 1 mL supernatant and mixed evenly, which was subsequently filtered through a 0.22 μm filter and passed through a Yilite UV3100 series HPLC instrument equipped with a Yilite C18 column (5 μm, 4.6 mm × 250 mm) and a refractive index detector (RID), and the injection volume was 10 μL. The mobile phase consisted of two eluents: eluent A was phosphate buffer (1 mM KH_2_PO_4_, pH 2.0), and eluent B was HPLC-grade acetonitrile. The isocratic program was 50% eluent A and 50% eluent B for 25 min. The flow rate was 1 mL/min, and the column temperature was 40°C.

LC–ESI–MS analysis was performed on a Waters Acuity UPLC BEH C18 column (1.7 μm, 100 mm × 2.1 mm) on a Waters ACQUITY UPLC ZQ2000 system (Waters Corporation, Milford, USA) at a flow rate of 1 mL/min via gradient elution of solvent A (methanol) and solvent B (0.01% acetic acid containing 5 mM ammonium acetate) as follows: 55% A (0–1.0 min), 55–62% A (1.0–2.6 min), and 62–80% A (2.6–11.4 min). The column temperature was 45°C, and the injection volume was 5 μL. The MS instrument was operated in ESI positive ion mode. The capillary voltage was optimized to 3.0 kV, and the cone voltage was 55 V. The source temperature was set to 120°C, the desolvation temperature was 300°C, the desolvation gas flow was set to 700 L/h, and the cone gas flow was 50 L/h.

The sample solution was subjected to liquid chromatography, and the degree of reaction conversion was determined by comparing the peak areas of CDCA, 7 K-LCA and UDCA in the sample solution. The conversion rate of UDCA is equal to the peak area of UDCA divided by the sum of the peak areas of UDCA, 7 K-LCA and CDCA, multiplied by 100%.

## 5 Results and discussion

### 5.1 Results for the construction of the engineered *Escherichia coli*

CDCA is well known to undergo a two-step reaction to synthesize UDCA (Figure 1B). The initial step involves the oxidation of CDCA by 7α-HSDH, resulting in the formation of 7 K-LCA. The subsequent step is the reduction of 7 K-LCA by 7β-HSDH, which ultimately generates UDCA. The initial reaction requires the involvement of the oxidative coenzyme I NAD^+^, and the subsequent reaction requires the involvement of the reductive coenzyme II NADPH. The cyclic regeneration of NAD^+^ and NADPH is typically achieved through the introduction of LDH and GDH, respectively, into the reaction system (Zheng et al., 2015; Song et al., 2023). The present study used four enzymes: 7α-HSDH (GenBank accession no. KXH01569) from *E. coli* (Yoshimoto et al., 1991), LDH (GenBank accession no. WP-011543503) from *Lactobacillus delbrueckii* (Bernard et al., 1991), 7β-HSDH (GenBank accession no. WP-015528793) from *Ruminococcus torques* (Zheng et al., 2015), and GDH (GenBank accession no. MDQ0804260.1) from *Priestia megaterium*. The gene sequences of the four enzymes were codon optimized in accordance with *E. coli* preferences and subsequently synthesized by GenScript Biotech Corporation.

#### 5.1.1 Results for the construction of engineered strains expressing a single enzyme

The primers listed in Supplementary Table S1 were used to amplify the LDH, 7α-HSDH, 7β-HSDH and GDH gene fragments, and the synthetic plasmids encoding the four enzymes were used as templates. As described in Methods Section 2.3, each of the gene fragments, which had been digested with two restriction enzymes, was separately ligated into the pET28a (+) plasmid, which had been digested with the same two enzymes. The recombinant plasmids pET28a-7α, pET28a-7β, pET28a-ldh, and pET28a-gdh were successfully constructed and separately transformed into the *E. coli* strain BL21 (DE3). The obtained positive transformants were verified with the primers listed in Supplementary Table S1. Agarose gel electrophoresis revealed that the sizes of the LDH, 7α-HSDH, 7β-HSDH, and GDH sequences were approximately 1,000 bp, 750 bp, 800 bp, and 800 bp, respectively, which were consistent with the theoretical values (Supplementary Figure S2). Four single-enzyme-expressing engineered strains were successfully constructed: UCA01 (carrying the plasmid pET28a-7α), UCA02 (carrying the plasmid pET28a-7β), UCA03 (carrying the plasmid pET28a-ldh), and UCA04 (carrying the plasmid pET28a-gdh).

#### 5.1.2 Results for the construction of engineered strains coexpressing two enzymes

The LDH/7α-HSDH gene combination and GDH/7β-HSDH gene combination were incorporated into MCS1 and MCS2 of the coexpression plasmids pACYCDuet-1, pETDuet-1 and pRSFDuet-1, respectively, to construct dual-enzyme coexpression plasmids. As detailed in Supplementary Table S1, the aforementioned four genes were amplified with the specified primers, and the resulting products, which had been digested with two restriction enzymes, were subsequently ligated in a sequential manner into the corresponding dual-enzyme coexpression plasmids, which had been digested with the same two enzymes, and the recombinant plasmids pACYCDuet-ldh-7α, pETDuet-ldh-7α, pRSFDuet-ldh-7α, pACYCDuet-gdh-7β, pETDuet-gdh-7β, and pRSFDuet-gdh-7β were successfully constructed (Supplementary Figure S1). Similarly, the recombinant plasmid pRSFDuet-7β-7α was constructed with the aforementioned methodology. The plasmids were subsequently transformed into the *E. coli* strain BL21(DE3), and the resulting transformants were validated by PCR, which revealed that the sizes of the genes were consistent with the theoretical values (Supplementary Figure S3). The construction of seven engineered strains with dual-enzyme coexpression was successful. These strains were designated UCA05 (carrying the plasmid pACYCDuet-ldh-7α), UCA06 (carrying the plasmid pETDuet-ldh-7α), UCA07 (carrying the plasmid pRSFDuet-ldh-7α), UCA08 (carrying the plasmid pACYCDuet-gdh-7β), UCA09 (carrying the plasmid pETDuet-gdh-7β), UCA10 (carrying the plasmid pRSFDuet-gdh-7β), and UCA11 (carrying the plasmid pRSFDuet-7β-7α).

#### 5.1.3 Results for the construction of engineered strains coexpressing four enzymes

Two types of strains coexpressing four enzymes were constructed. The first type contained two plasmids, with the two plasmids coexpressing LDH/7α-HSDH and GDH/7β-HSDH. This strain was designated the four-enzyme dual-plasmid combination strain. The second type contained one plasmid, with the four enzymes integrated into the same plasmid for coexpression. This strain was designated the four-enzyme single-plasmid strain.

Construction of four-enzyme dual-plasmid combination strains: Plasmids were extracted from the constructed UCA05-UCA10 strains, combining four enzymes with different resistances, and then transformed into BL21(DE3) cells. The construction of six strains coexpressing four enzymes from two plasmids was successfully completed. The resulting strains were designated UCA12 (carrying the plasmids pACYCDuet-ldh-7α and pRSFDuet-gdh-7β), UCA13 (carrying the plasmids pACYCDuet-ldh-7α and pETDuet-gdh-7β), UCA14 (carrying the plasmids pRSFDuet-ldh-7α and pACYCDuet-gdh-7β), UCA15 (carrying the plasmids pRSFDuet-ldh-7α and pETDuet-gdh-7β), UCA16 (carrying the plasmids pETDuet-ldh-7α and pACYCDuet-gdh-7β), and UCA17 (carrying the plasmids pETDuet-ldh-7α and pRSFDuet-gdh-7β) (Supplementary Table S3).

Construction of four-enzyme single-plasmid strains: The gdh-7β, ldh-7α, gb and la genes were amplified with the primers listed in Supplementary Table S1. The gdh-7β/ldh-7α gene combinations were subsequently digested with two restriction enzymes and ligated into the coexpression plasmids pACYCDuet-1, pETDuet-1 and pRSFDuet-1, which were previously digested with the same two enzymes. The recombinant plasmids pACYCDuet-gdh-7β-ldh-7α, pETDuet-gdh-7β-ldh-7α, and pRSFDuet-gdh-7β-ldh-7α were successfully constructed. Similarly, the gene combinations gb/la, gb/ldh-7α and gdh-7β/la were digested with two restriction enzymes and separately ligated into the coexpression plasmid pRSFDuet-1. Recombinant plasmids containing genes with promoter deletions, namely, pRSFDuet-gb-la, pRSFDuet-gb-ldh-7α, and pRSFDuet-gdh-7β-la, were successfully constructed. The recombinant plasmids, which had been successfully constructed, were subsequently transformed into BL21(DE3) cells. The target genes were subjected to further verification by PCR with the primers listed in Supplementary Table S1 (Supplementary Figures S4, S5). Six four-enzyme single-plasmid coexpression strains were successfully constructed. These strains were designated UCA18 (carrying the plasmid pACYCDuet-gdh-7β-ldh-7α), UCA19 (carrying the plasmid pETDuet-gdh-7β-ldh-7α), UCA20 (carrying the plasmid pRSFDuet-gdh-7β-ldh-7α), UCA21 (carrying the plasmid pRSFDuet-gb-la), UCA22 (carrying the plasmid pRSFDuet-gb-ldh-7α), and UCA23 (carrying the plasmid pRSFDuet-gdh-7β-la).

### 5.2 Analyses of the heterologous expression and activity of the recombinant enzymes

All the successfully constructed strains were fermented in shaker flasks, and the expression and activity of enzymes in each strain were assessed after 4 h of induction at 30°C with 0.5 mM IPTG.

#### 5.2.1 Detection of enzyme expression and activity in engineered strains expressing single enzymes

**Figure 3:**
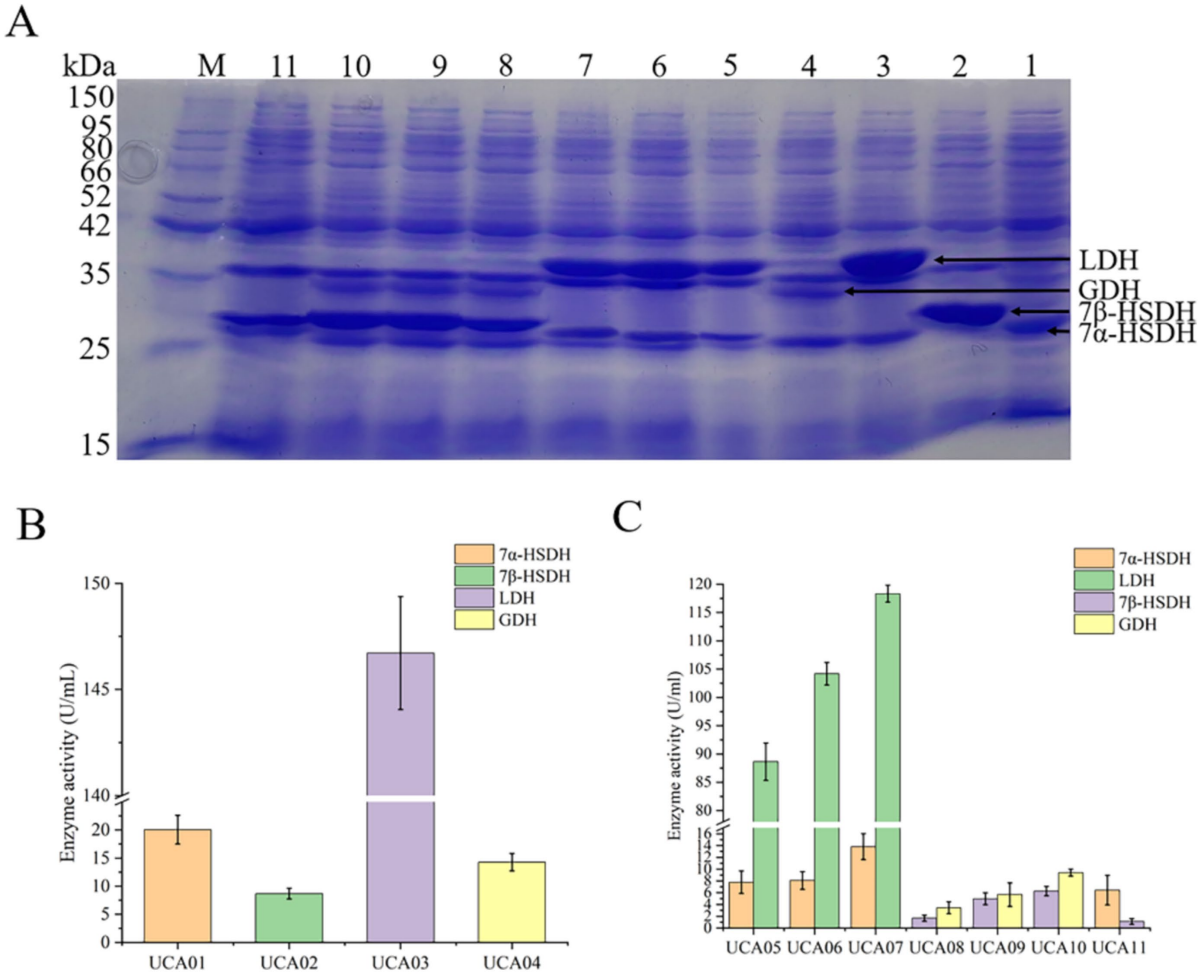
Expression and activity analysis of 7α-hydroxysteroid dehydrogenase (7α-HSDH), lactate dehydrogenase (LDH), 7β-hydroxysteroid dehydrogenase (7β-HSDH) and glucose dehydrogenase (GDH) in recombinant *E. coli* strains UCA01-UCA11. **(A)** SDS-PAGE analysis of 7α-HSDH, LDH, 7β-HSDH, and GDH expressed respectively, in *E. coli* UCA01 (lane 1), UCA02 (lane 2), UCA03 (lane 3), UCA04 (lane 4), UCA05 (lane 5), UCA06 (lane 6), UCA07 (lane 7), UCA08 (lane 8), UCA09 (lane 9), UCA10 (lane 10), UCA11 (lane 11); lane M, protein ladder. **(B)** Enzyme activity of 7α-HSDH, 7β-HSDH, LDH, and GDH expressed in *E. coli* UCA01-UCA04. **(C)** Enzyme activity of 7α-HSDH, LDH, 7β-HSDH, and GDH expressed in *E. coli* UCA05-11.

UCA01, UCA02, UCA03, and UCA04 expressed 7α-HSDH, 7β-HSDH, LDH, and GDH, respectively. The theoretical molecular weights of the four enzymes were 26 kDa, 28 kDa, 37 kDa, and 29 kDa, respectively. The results of the SDS–PAGE analysis indicated that the four enzymes were expressed normally, with apparent molecular weights consistent with the theoretical values. The enzyme activity test was conducted using a crude enzyme mixture. The enzyme activities were 20.1 U/mL, 8.7 U/mL, 146.7 U/mL, and 14.3 U/mL, respectively (Figure 3).

#### 5.2.2 Detection of enzyme expression and activity in engineered strains coexpressing two enzymes

UCA05, UCA06, and UCA07 coexpressed LDH and 7α-HSDH, and UCA08, UCA09, and UCA10 coexpressed GDH and 7β-HSDH. Among these strains, the UCA07 strain presented the highest activities of the LDH and 7α-HSDH enzymes, reaching 118.3 U/mL and 13.8 U/mL, respectively, and the UCA10 strain presented the highest activities of the GDH and 7β-HSDH enzymes, reaching 9.4 U/mL and 6.3 U/mL, respectively. The recombinant *E. coli* UCA11 strain coexpressed 7β-HSDH and 7α-HSDH, with enzyme activities of 1.1 U/mL and 6.5 U/mL, respectively. All the strains coexpressing two enzymes were capable of expressing the target proteins; nevertheless, a discrepancy in the level of expression was observed (Figure 3). In this study, the three most utilized plasmids for coexpression of genes in *E. coli*, namely, pACYCDuet-1, pETDuet-1 and pRSFDuet-1, were selected for the coexpression of two enzymes. The three plasmids contain distinct replicons: pACYCDuet-1 carries the p15A replicon, pETDuet-1 carries the pBR322 replicon, and pRSFDuet-1 carries the RSF replicon. Their copy numbers within cells were observed to be 10, 40, and 100, respectively (Wu et al., 2013). The distinct replicons of the plasmids resulted in varying copy numbers, which subsequently influenced the levels of gene expression (Nordén et al., 2011; Standley et al., 2019; Mehta et al., 2021). The expression levels of proteins encoded by high-copy-number plasmids are typically greater than those of proteins encoded by low-copy-number plasmids (Carrier et al., 1998; Jahn et al., 2016). Consequently, adjusting the plasmid copy number is a common strategy for regulating protein expression levels (Zhou et al., 2017; Sun et al., 2023; Hu and Zhang, 2024). Among the strains coexpressing two enzymes used in this study, UCA07 coexpressed LDH and 7α-HSDH with the highest expression levels and enzyme activities, and UCA10 coexpressed GDH and 7β-HSDH with the highest expression levels and enzyme activities. Both strains carried the high-copy-number plasmid pRSFDuet-1 (Supplementary Figure S1).

#### 5.2.3 Detection of enzyme expression and activity in engineered strains coexpressing four enzymes

**Figure 4:**
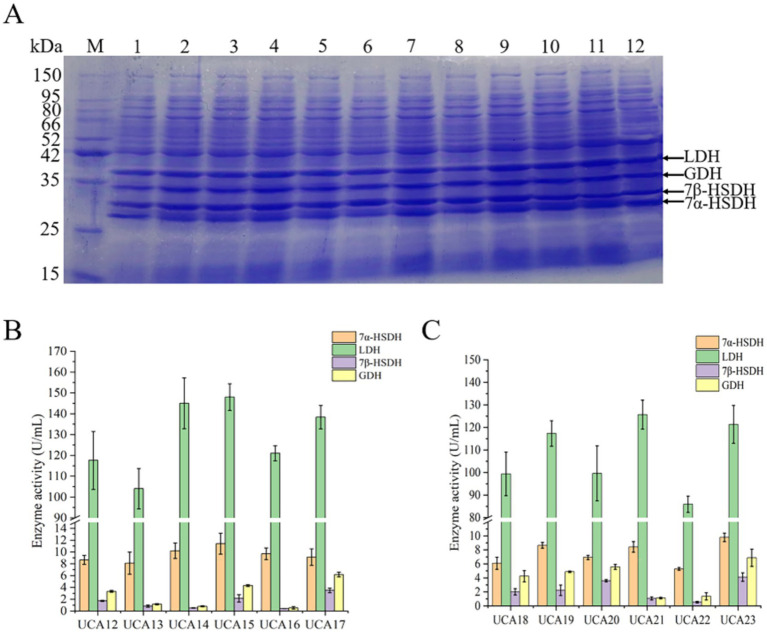
Expression and activity analysis of 7α-HSDH, LDH, 7β-HSDH and GDH in recombinant *E. coli* strains UCA12-UCA23. **(A)** SDS-PAGE analysis of 7α-HSDH, LDH, 7β-HSDH, and GDH expressed respectively, in *E. coli* UCA12 (lane 1), UCA13 (lane 2), UCA14 (lane 3), UCA15 (lane 4), UCA16 (lane 5), UCA17 (lane 6), UCA18 (lane 7), UCA19 (lane 8), UCA20 (lane 9), UCA21 (lane 10), UCA22 (lane 11), UCA23 (lane 12); lane M, protein ladder. **(B)** Enzyme activity of 7α-HSDH, LDH, 7β-HSDH, and GDH expressed in *E. coli* UCA12-UCA17. **(C)** Enzyme activity of 7α-HSDH, LDH, 7β-HSDH, and GDH expressed in *E. coli* UCA18-UCA23.

In this study, two modes of dual-plasmid coexpression strains (UCA12-UCA17) and single-plasmid coexpression strains (UCA18-UCA23) were used to simultaneously express GDH, 7β-HSDH, LDH, and 7α-HSDH in a single strain. The results of the SDS–PAGE analysis revealed that all the constructed strains were successfully capable of expressing the four enzymes (Figure 4). A total of six dual-plasmid coexpression strains were constructed and designated UCA12-UCA17 (Supplementary Figure S1). Among the strains, UCA15 presented the highest activities of the LDH and 7α-HSDH enzymes, reaching 148.3 U/mL and 11.4 U/mL, respectively. UCA17 exhibited the highest activities of the GDH and 7β-HSDH enzymes, reaching 6.2 U/mL and 3.5 U/mL, respectively (Figure 4B). The dual-plasmid coexpression system was comprised of two distinct plasmids, each carrying a different resistance gene. Two types of antibiotics must be added to the culture medium to maintain screening pressure, which places considerable strain on the normal growth and metabolic processes of the strain (Zhou et al., 2017). The growth rate of the dual-plasmid coexpression strain was significantly slower than that of the single-plasmid coexpression strain. The strains coexpressing the four enzymes from a single plasmid were validated with three plasmids (pACYCDuet-1, pETDuet-1 and pRSFDuet-1) in a preliminary experiment (the results of which are not presented here). The pRSFDuet-1 plasmid presented the most effective expression, and the position and number of promoters were subsequently optimized based on the pRSFDuet-1 plasmid. Six single-plasmid coexpression strains were constructed and designated UCA18-UCA23 (Supplementary Figure S1). SDS–PAGE and enzyme activity assays revealed that UCA21 presented the highest enzyme activity for LDH, with an enzyme activity of 125.7 U/mL, whereas UCA23 presented the highest activities for 7α-HSDH, GDH and 7β-HSDH, with enzyme activities of 9.8 U/mL, 6.9 U/mL and 4.1 U/mL, respectively (Figure 4C). The position and number of promoters significantly affect gene expression. Genes near the promoter typically exhibit considerably higher expression levels than those located at greater distances. The number of promoters should also match the expression of the target gene. An excess number of promoters exerts undue stress on cellular metabolism, which in turn affects gene expression levels (Juminaga et al., 2012; Liu et al., 2019). The results of this experiment corroborate this assertion. For example, the 7β-HSDH gene was near the promoter, resulting in elevated 7β-HSDH enzyme activity. Conversely, when the gene was located farther from a promoter, enzyme activity was diminished.

### 5.3 Whole-cell biotransformation

#### 5.3.1 Whole-cell biotransformation of different engineered *Escherichia coli* combinations

##### 5.3.1.1 Combinations of engineered strains expressing a single enzyme

Strains UCA01, UCA02, UCA03, and UCA04 were combined for the whole-cell catalysis of CDCA. The substrate, auxiliary ingredients and four bacterial sludges were introduced in a sequential manner to the 50 mL reaction system. After the reaction, the conversion rate of UDCA was determined to be 5%. When catalyzed by engineered strains expressing individual enzymes, CDCA initially enters UCA01 cells to catalyze the synthesis of 7 K-LCA, with the conversion rate of this step reaching 99%. Subsequently, 7 K-LCA must be transported from UCA01 to the extracellular space (Rudroff, 2019). Furthermore, 7 K-LCA is susceptible to precipitation in buffer, which precludes its continued participation in the reaction. Consequently, only a negligible quantity of solubilized 7 K-LCA can enter UCA02 cells to proceed with the reaction, resulting in an exceedingly low conversion rate of 7 K-LCA to UDCA. The synthesis of UDCA from CDCA was catalyzed by the combination of engineered strains expressing individual enzymes, which ultimately resulted in a low conversion rate of UDCA. This outcome was due to the continuous mass transfer of the substrate CDCA and the intermediate 7 K-LCA from cell to cell and necessitated active transport for entry into and exit from the cell. This process resulted in elevated mass transfer resistance (Lin and Tao, 2017). Four equal quantities of bacterial sludges were broken open, and CDCA was transformed to UDCA by the crude enzyme mixture under identical conditions. The conversion rate of UDCA reached 85%. This finding indicates that the spatial barrier inherent to the combination of engineered strains expressing individual enzymes has a considerable influence on the transformation process.

##### 5.3.1.2 Combinations of engineered strains coexpressing two enzymes

**Figure 5:**
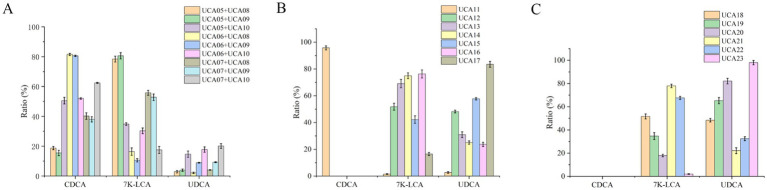
The ratio of chenodeoxycholic acid (CDCA), 7-ketolithocholic acid (7 K-LCA) and ursodeoxycholic acid (UDCA) following whole-cell catalysis reaction for 4 h by dual-enzyme coexpression strains and two types of four-enzyme coexpression strains. **(A)** Whole-cell catalysis reaction by combinations of dual-enzyme coexpression strains. **(B)** Whole-cell catalysis reaction by four-enzyme coexpression strains containing two plasmids. **(C)** Whole-cell catalysis reaction by four-enzyme coexpression strains containing one plasmid.

When the engineered strains expressing both LDH/7α-HSDH and GDH/7β-HSDH were combined for whole-cell catalysis, nine reaction combinations could be formed (Figure 5A). The highest conversion rate of UDCA was achieved when it was catalyzed by the whole-cell combination of UCA07 and UCA10, which achieved the complete conversion of CDCA to 7 K-LCA. Nevertheless, the conversion rate of 7 K-LCA to UDCA was merely 20% (Figure 5A). The low conversion of 7 K-LCA to UDCA can be attributed to the same factors as those observed in the combination of engineered strains expressing individual enzymes. Specifically, 7 K-LCA must be secreted from cells that coexpress LDH and 7α-HSDH into the extracellular reaction system and subsequently enter cells that coexpress GDH and 7β-HSDH to synthesize UDCA. Although the intracellular–extracellular–intracellular mass transfer process is complete, the same issue persists: high resistance to mass transfer and a significant loss of intermediate products (Lin and Tao, 2017). When equal amounts of the aforementioned cells were broken open and the catalytic reaction was conducted with an enzyme mixture, the UDCA conversion rate reached 80% under identical conditions.

##### 5.3.1.3 Engineered strains coexpressing four enzymes

When whole-cell catalysis was performed by the dual-plasmid coexpression strains UCA12-UCA17, the highest UDCA conversion rate of 83% was observed for UCA17. Furthermore, when whole-cell catalysis was performed with the single-plasmid coexpression strains UCA18-UCA23, the highest UDCA conversion rate of 98% was observed for UCA23 (Figures 5B,C). The two types of four-enzyme coexpression strains exhibited superior conversion of CDCA to UDCA in comparison to both the single-enzyme expression strains and the dual-enzyme coexpression strains, with both exceeding 80%. Furthermore, the analysis revealed a positive correlation between the conversion rates of the four-enzyme coexpression strains and the expression and activity of 7β-HSDH. The dual-plasmid coexpression strain UCA17 and the single-plasmid coexpression strain UCA23 presented the highest expression level of 7β-HSDH, accompanied by high enzyme activity, resulting in a high conversion rate of UDCA. This result occurred because of the four enzymes utilized, LDH and 7α-HSDH exhibited high enzyme activities, whereas GDH displayed good stability. In contrast, 7β-HSDH has the lowest enzyme activity and stability (Huang et al., 2023; Song et al., 2023). Consequently, 7β-HSDH can be considered the overall rate-limiting enzyme. Furthermore, UCA11 coexpressed 7α-HSDH and 7β-HSDH, whereas LDH and GDH were not expressed. This strain exhibited a conversion rate of only 2.7% for UDCA, which suggests that the coenzyme regeneration system significantly influences the reaction. The engineered strains coexpressing the four enzymes catalyzed the reaction in the same cell, thereby completing the two-step reaction. This process did not involve the intermediate product 7 K-LCA being shuttled between cells, and the problem of ‘intercellular mass transfer resistance’ did not occur. This process greatly improved the conversion efficiency (Wachtmeister and Rother, 2016; Lin and Tao, 2017). The strain with the most effective catalysis among the engineered strains coexpressing the four enzymes was UCA23 (Figures 5B,C). This strain carried a high-copy-number plasmid, pRSFDuet-1, which contained a sequence to express the gdh-7β-la gene and three promoters. The GDH and 7β-HSDH genes were each preceded by a single promoter. This approach ensures high transcription of the two genes. In contrast, LDH and 7α-HSDH have high enzyme activities; thus, sharing a single promoter can also satisfy the enzyme activity requirements of both enzymes. From the perspective of matching the enzyme activities of each enzyme involved in the catalytic reaction, UCA23 was identified as the optimal catalytic strain.

**Figure 6:**
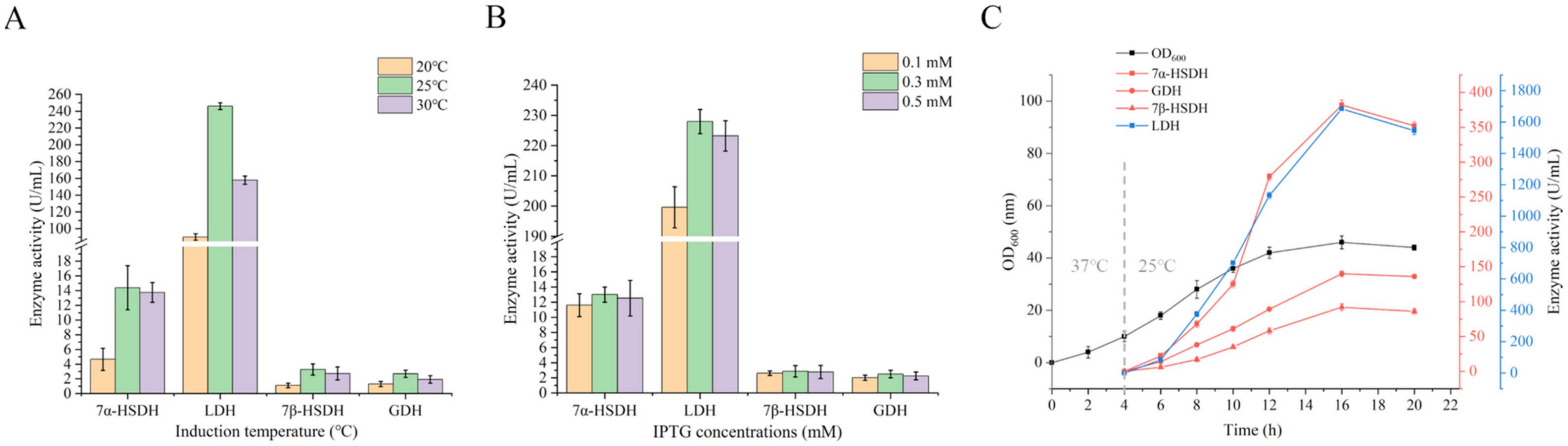
Effect of induction parameters on 7α-HSDH, LDH, 7β-HSDH, and GDH enzyme activities for UCA23 strain. **(A)** Effect of induction temperatures on the activity of 7α-HSDH, LDH, 7β-HSDH, and GDH. **(B)** Effect of IPTG concentration on the 7α-HSDH, LDH, 7β-HSDH, and GDH activity. **(C)** The time course of the UCA23 cell culture (OD600) and enzyme activity.

UCA23 was fermented in a 5 L fermenter to obtain the optimal catalytic cell, which was subsequently used for the reaction. Following this step, the optimal catalytic conditions were identified. The optimal induction temperature and IPTG concentration for UCA23 were previously determined to be 25°C and 0.3 mM, respectively, in shaker flasks (Figures 6A,B). The optimal induction temperature and IPTG concentration were sed in the fermentation of UCA23 in a 5 L fermenter. When UCA23 was cultured at 37°C for 4 h, induction was started by cooling to 25°C and adding 0.3 mM IPTG. Following a 12 h induction period, the OD_600_ value of the fermentation broth reached 46, and the expression and activities of the four enzymes in the fermentation broth reached their highest levels, with the enzyme activities of 7α-HSDH, 7β-HSDH, LDH, and GDH reaching 381. 2 U/mL, 90.1 U/mL, 1685.8 U/mL and 142.6 U/mL, respectively (Figure 6C). At this point, the UCA23 cells were harvested for subsequent experiments aimed at optimizing the catalytic conditions.

#### 5.3.2 Optimization of whole-cell catalytic conditions for UCA23

The optimal catalytic conditions for UCA23 were determined in a 50 mL reaction system. The effects of the reaction temperature (20–40°C), pH (6.0–9.5), PBS concentration (25–150 mM), NADP^+^ concentration (0–50 μM), 5% organic solvents, and 0.5% surfactants on whole-cell catalysis were investigated.

**Figure 7:**
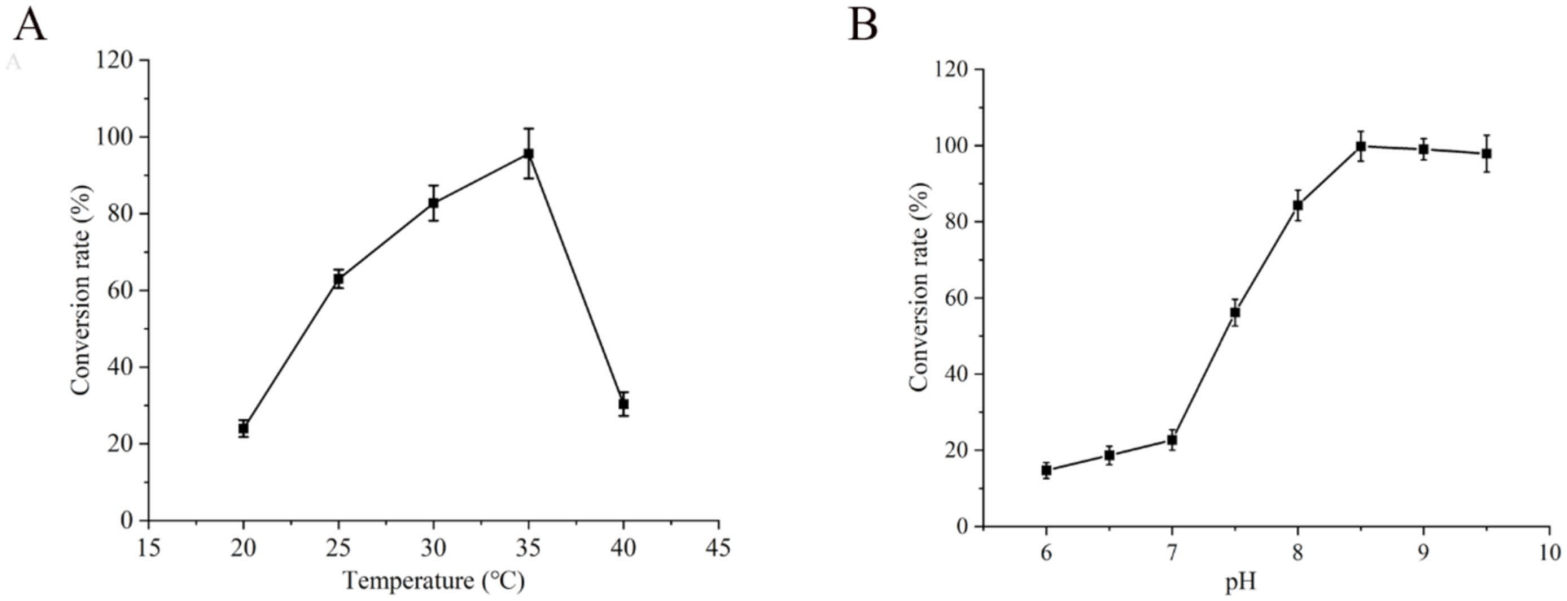
Effect of **(A)** temperatures and **(B)** pHs on conversion rate of CDCA to UDCA.

The effect of the reaction temperature on whole-cell catalysis was examined while keeping the other reaction parameters constant. The highest conversion rate of UDCA was achieved at a reaction temperature of 35°C, with a conversion rate of 96% (Figure 7A). The conversion rate of UDCA was positively correlated with temperature within the range of 20°C–35°C. However, when the temperature exceeded 35°C, the UDCA conversion rate gradually decreased as the temperature increased, decreasing to 30% when the temperature reached 40°C. A reduction in temperature resulted in a decrease in enzyme activity, which in turn led to a reduction in the UDCA conversion rate. Furthermore, an increase in temperature resulted in an increase in enzyme activity; however, this increased temperature also affected the stability of the enzymes. Lower temperatures are conducive to maintaining cell stability, although at the expense of enzyme activity. Conversely, higher temperatures may increase enzyme activity, although they are not conducive to maintaining cell integrity over time (Farewell and Neidhardt, 1998; Lin and Tao, 2017; Xu et al., 2019). In this study, 35°C was identified as the equilibrium point that would maintain the stability of the UCA23 enzyme activity and a high conversion rate.

Figure 7B illustrates the effect of pH on whole-cell catalysis, showing that the highest UDCA conversion rate of 99% was achieved at pH 8.5 within the pH 6.0–9.5 assay range. In the pH range of 6.0–7.0, the conversion rate slightly increased from 15 to 23% with increasing pH. In contrast, within the pH range of 7.0–8.5, the UDCA conversion rate notably increased to 99% from 23%. However, above pH 8.5, the conversion rate tended to decrease. CDCA is soluble in alkaline solutions, yet it is unable to dissolve completely under neutral or acidic conditions (Igimi and Carey, 1980). The undissolved CDCA is unable to enter the cell for effective transformation, resulting in a low conversion rate of UDCA. As the pH increases, the solubility of CDCA increases, resulting in a corresponding increase in the UDCA conversion rate. However, if the pH exceeds the optimal range, a highly alkaline environment can cause cellular instability, such as autolysis problems, which in turn affects whole-cell catalysis (Parhad and Rao, 1974).

**Figure 8:**
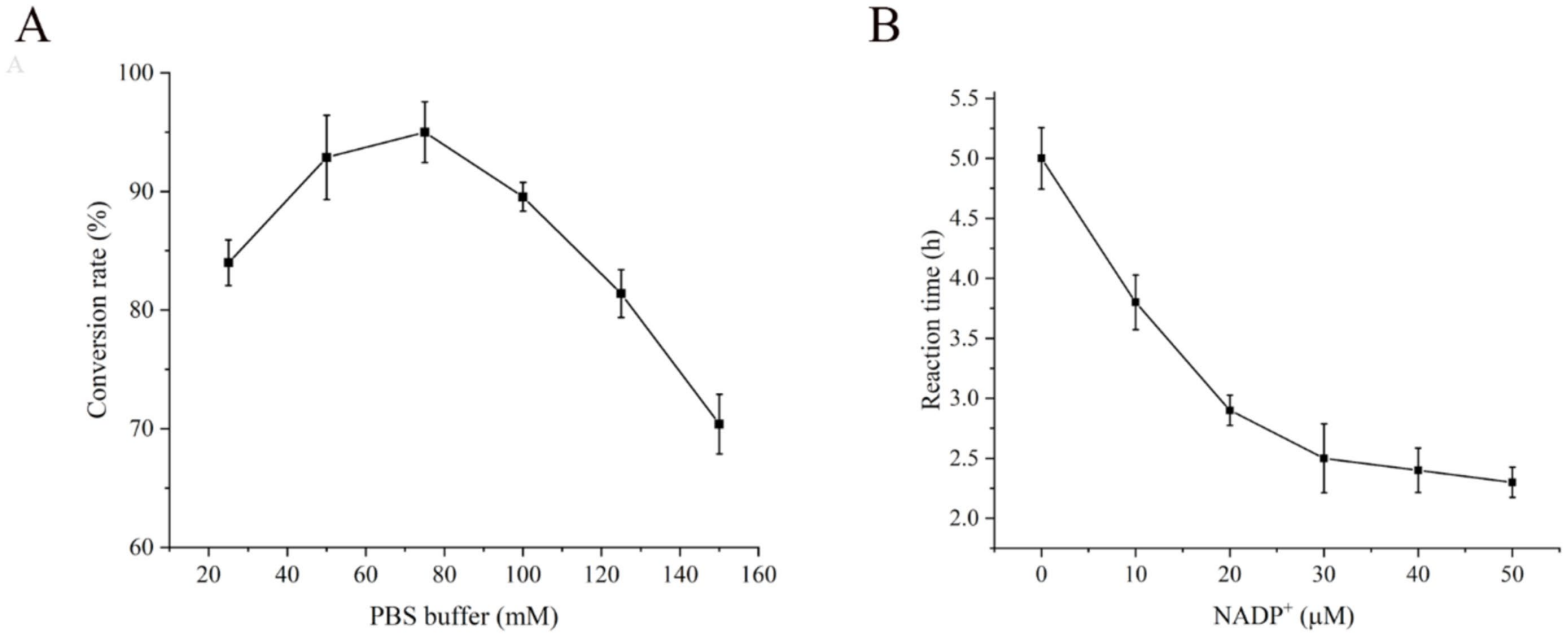
Effect of **(A)** PBS buffer on conversion rate and **(B)** NADP^+^ on conversion efficiency of CDCA to UDCA.

The function of the PBS buffer is to regulate the balance of salt ions and the pH value. Different PBS buffer concentrations influence the normal transport of intracellular and extracellular substances, solubilization of substrates, enzyme activity and stability (Xu et al., 2019). The objective of this study was to investigate the effects of various PBS buffer concentrations (25–150 mM) on the conversion of CDCA to UDCA. The results indicated that when the PBS buffer concentration in the reaction system was 75 mM, the highest UDCA conversion rate of 95% was achieved (Figure 8A). However, the UDCA conversion rate decreased when the PBS buffer concentration was either lower or higher than 75 mM. The utilization of PBS buffer as the solution system for the reaction enables the dissolution of the substrate and intermediate while maintaining the pH stability of the entire reaction system, thus ensuring the normal progression of the reaction.

The four enzymes in this study catalyzed the synthesis of UDCA from CDCA, and LDH and GDH were used for the cyclic regeneration of the coenzymes NAD^+^ and NADPH, respectively. The *E. coli* cells themselves contain approximately 20 times more NAD(H) than NADP(H) (Bennett et al., 2009). We supplemented the system with 0–50 μM NADP^+^ to coordinate the two-step reaction and verified the effect of the coenzyme NADP^+^ on transformation. The experimental results revealed that the addition of exogenous NADP^+^ markedly increased the conversion efficiency. The reaction used the endogenous NADP^+^ within the cells to achieve complete conversion of CDCA to UDCA for 5 h, without the necessity for additional NADP^+^ supplementation. As the concentration of NADP^+^ increased from 0 to 50 μM, the time required for the complete conversion of CDCA decreased from 5.0 h to 2.3 h (Figure 8B). Accordingly, from the standpoint of production, the efficiency of production can be increased by the addition of an optimal quantity of NADP^+^ to the reaction system.

**Figure 9:**
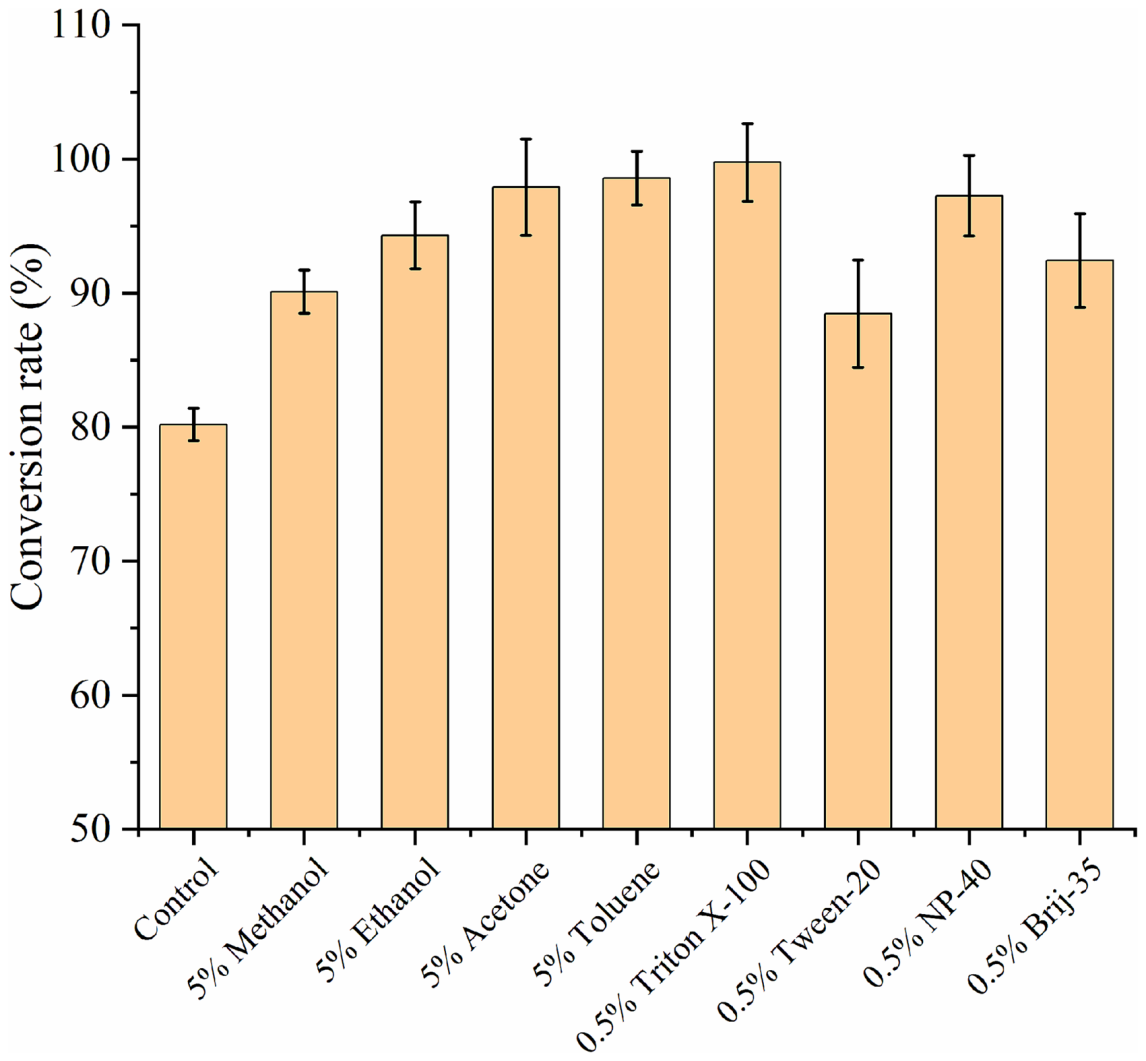
Effect of organic solvents and surfactants on conversion rate of CDCA to UDCA.

An increase in cell membrane permeability can markedly increase the rate of intracellular and extracellular substance exchange, thereby accelerating the whole-cell catalytic reaction and improving the UDCA conversion rate (Yang et al., 2019). The use of low concentrations of organic solvents and surfactants has been shown to be less toxic to cells, and they have the potential to increase the permeability of *E. coli* cell membranes and increase the solubility of substrates and products. The effects of 5% organic solvents (methanol, ethanol, acetone, and toluene) and 0.5% surfactants (Triton X-100, Tween-20, NP-40, and Brij-35) on the transformation of CDCA to UDCA were examined. The control group, which did not include the addition of organic solvents or surfactants, presented a UDCA conversion rate of 80%. The UDCA conversion rate was greater than 90% when 5% organic solvents were introduced. The highest UDCA conversion rate of 98% was achieved with the addition of 5% toluene. Following the addition of 0.5% surfactant, the best outcome was observed with 0.5% Triton X-100, resulting in a UDCA conversion rate of 99.5% (Figure 9). In comparison, methanol (UDCA conversion rate of 90%) and Tween-20 (UDCA conversion rate of 88%) exhibited comparatively lower efficacy. In general, both organic solvents and surfactants were conducive to the whole-cell conversion of CDCA.

**Figure 10:**
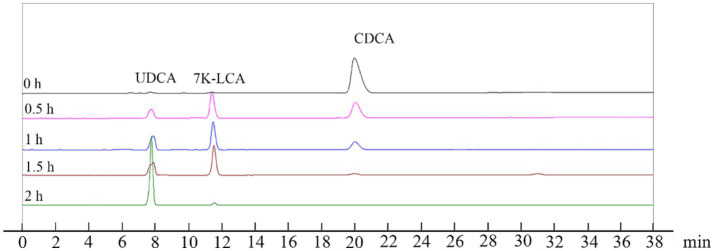
HPLC analysis of CDCA, 7 K-LCA, and UDCA after whole-cell catalysis reaction for 0 h, 0.5 h, 1 h, 1.5 h, and 2 h. The ratios of the CDCA, 7 K-LCA and UDCA peak areas for each HPLC analysis were 100: 0:0, 46:39:15, 24:47:29, 5:59:36, and 0:1:99, respectively.

The reaction system was expanded to 2 L using the optimized reaction conditions (reaction temperature of 35°C, pH 8.5, 50 μM NADP^+^, and 0.5% Triton X-100) and contained 100 mM CDCA, 150 mM sodium pyruvate and 150 mM glucose to validate the conversion effect of UCA23. The conversion rate of UDCA from 100 mM CDCA reached 99% after 2 h (Figure 10). Notably, the majority of enzymatic syntheses of UDCA tend to fail to achieve complete conversion of the substrate because of the presence of a reaction equilibrium (Zheng et al., 2015; Zheng et al., 2017b). In this study, the utilization of the UCA23 strain appeared to disrupt the equilibrium, ultimately allowing a conversion rate of 99% from CDCA to UDCA to be achieved, which is an almost complete conversion. A possible explanation for this phenomenon is that the generated UDCA is secreted outside the cell, which results in spatial separation between the substrate and the product. This separation drives the reaction to continue in the direction of product formation (Monti et al., 2009). A comparison of the conversion efficiency of UCA23 with the results reported in the literature revealed that the whole-cell conversion of UCA23 exhibited superior performance in terms of the conversion rate, conversion efficiency and substrate loading capacity (Table 1). An LC–ESI–MS analysis was conducted to further confirm the identity of the product generated by UCA23 transformation. The retention time of UDCA was 7.9 min (Figure 10), and ESI–MS analysis was conducted on the UDCA components isolated by HPLC. The MS peaks indicated that the molecular weight of the product was 392.58. This result confirmed that the final product was UDCA (C_24_H_40_O_4_, molecular weight: 392.58; [M-2H_2_O + H]^+^ at m/z 357.28, [2 M + H]^+^ at m/z 786.10) (Supplementary Figure S6).

**Table 1:** The biotransformation results of CDCA to UDCA.

Entry	Substrate load (mM)	NAD^+^ (mM)	NADP^+^ (mM)	Reaction time (h)	Conversion rate (%)	Space–time yield (g·L^−1^·d^−1^)	Reaction volume (mL)	References
1	10	0.5	0.5	2	98	446.3	1	Zheng et al. (2015)
2	10	0.15	0.15	1.5	99	62.1	5	Chen et al. (2019)
3	40	0.15	0.15	24	60	9.4	5	Chen et al. (2019)
4	100	0.5	0.5	5.3	80	141	10	Zheng et al. (2017b)
5	12	0.2	0.2	20	76	–	1 × 10^3^	Pedrini et al. (2006)
6	25	0.3	0	1	98.5	192	500	Wang et al. (2023)
7	100	0	0.05	2	99	465.7	2 × 10^3^	This study

To compare the changes in NAD^+^/NADH and NADP^+^/NADPH before and after the whole-cell catalyzed reaction, the UCA11 strain, which does not express LDH and GDH, was utilized. The strain was assessed with the NAD(P)/NAD(P)H kit, and it was ascertained that, prior to whole-cell catalysis, NAD^+^/NADH and NADP^+^/NADPH in the UCA11 strain itself remained at 35.465 and 0.014, respectively. Following whole-cell catalysis, the level of NAD^+^/NADH in UCA11 decreased to 0.882, owing to 7α-HSDH consuming NAD^+^ to generate NADH. This process was unable to rapidly regenerate NAD^+^ by utilizing the cell’s own coenzymatic homeostasis mechanism. Concurrently, the level of NADP^+^/NADPH increased to 28.215, and 7β-HSDH utilized NADPH to generate NADP^+^. Consequently, the cell itself was similarly unable to rapidly achieve coenzyme homeostasis. In contrast, the addition of LDH, GDH and related substrates, followed by whole-cell catalysis, enabled UCA11 to rapidly regenerate coenzymes. The level of NAD^+^/NADH increased to 31.338, and the level of NADP^+^/NADPH in UCA11 decreased to 0.025. Thus, it can be concluded that the introduction of LDH and GDH ensured the rapidity of the reaction and increased the efficiency of the reaction conversion.

#### 5.3.3 Results of the analysis of UCA23 cell recycling for catalysis

**Figure 11:**
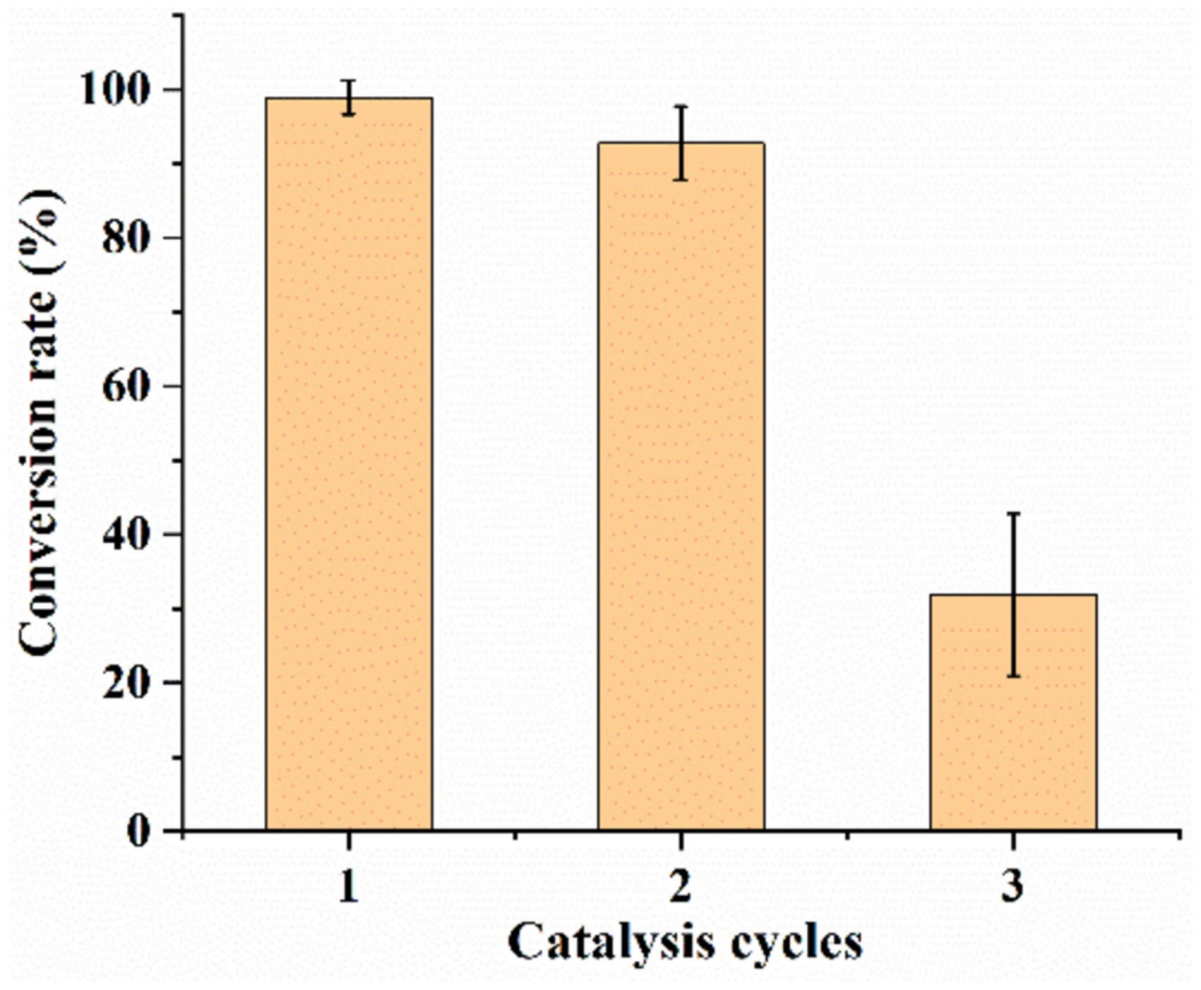
Repeated use of cells to synthesize UDCA from CDCA.

The sequential catalysis of multiple batches of substrates by UCA23 cells was investigated based on the reusability of the cells in a general whole-cell catalytic process (Figure 11). Under the optimal reaction conditions, the conversion rate of the whole-cell-catalyzed CDCA synthesis of UDCA was 99% in the first-round. After the reaction, the centrifugation and collection of cells for the second-round of the reaction resulted in a UDCA conversion rate of 93%. After the reaction and recollection of cells for the third-round, the UDCA conversion rate was 32%. The loss of both cellular and enzyme activity that can occur during the utilization of the cells and the physical manipulation of the cells during the collection of the cells can result in damage to the cells. These factors were the causes of the gradual decrease in the conversion rate of UDCA when UCA23 cells were used repeatedly. Nevertheless, this issue can be addressed by introducing fresh cells. In this study, after the addition of 40% fresh whole cells to the cells collected in the third-round, the UDCA conversion rate was restored to a level comparable to that of the first-round reaction.

## 6 Conclusion

In this study, three distinct whole-cell catalytic systems for the synthesis of UDCA were constructed: a combination of engineered strains expressing a single enzyme, a combination of engineered strains coexpressing two enzymes, and a catalytic system consisting of an engineered strain coexpressing all four enzymes. All the constructed strains successfully expressed the four enzymes necessary for the synthesis of UDCA from CDCA. The most effective catalytic strain, UCA23, was observed to achieve a 99% conversion rate of UDCA from 100 mM CDCA after 2 h under optimal conditions, which has industrial application value. Unlike free enzyme catalysis, whole-cell-catalyzed CDCA synthesis of UDCA in this study was conducted with no or minimal addition of coenzymes to the reaction system, thereby reducing the cost of the reaction. Furthermore, the system can use a substrate concentration of 100 mM, which enhances the efficiency of the synthesis. Compared with free enzymes, which are directly exposed to the reaction system, the intracellular environment plays a crucial role in protecting the enzyme within the cell and prolonging its lifespan. Moreover, the utilization of whole-cell catalysis facilitates the subsequent removal of bacterial proteins and reduces the burden on the subsequent purification process in industrial production. This approach is more conducive to realizing the green production of UDCA.

## Data Availability

The datasets presented in this study can be found in online repositories. The names of the repository/repositories and accession number(s) can be found in the article/Supplementary material.
