# Supplementary material for: Cost-effective whole-cell biosynthesis of ursodeoxycholic acid using engineered Escherichia coli with a multienzyme cascade
Source: Front Microbiol. 2025 Jan 22;16:1538237. doi: 10.3389/fmicb.2025.1538237 (PMC11808682; doi:10.3389/fmicb.2025.1538237)
Supplement: Supplementary file 1 [file Supplementary_file_1.docx]

Supplementary Material

**Supplementary Table 1.** Primers constructed for the expressing plasmids

| **Primer** | **Nucleotide sequence (5**′**-3**′**)** | **Restriction enzyme cutting site or overlapping base** | **Number of bases** | **Notes** |
| --- | --- | --- | --- | --- |
| LDH-F | CATGCCATGGGCATGACAAAAAT | *Nco* I | 23 |  |
| LDH-R | CCAAGCTTTTAACCAACTTTCACCGGC | *Hind* III | 27 |  |
| 7α-F | CGCCATATGTTCAACAGCGACAAC | *Nde* I | 24 |  |
| 7α-R | CGCTCGAGTTAGTTCAGTTCTTGCACGCC | *Xho* I | 29 |  |
| GDH-F | CTAGCCATGGGCATGCCAGCTCCCTATAA | *Nco* I | 29 |  |
| GDH-R | CCCAAGCTTTTACGAACTCCAGTTGTTC | *Hind* III | 28 |  |
| 7β-F | CGCCATATGAACCTGCGTGAGAA | *Nde* I | 23 |  |
| 7β-R | CGCTCGAGTTAGTTGTTGCTATAGAAGCTACC | *Xho* I | 32 |  |
| GB-1 | **aaggagatatacaT**ATGAACCTGCGTGAGAAG | **Overlapping base** | 32 | Removal of the T7 promoter preceding 7β and the T7 promoter preceding 7α |
| GB-2 | **Atgtatatctcctt**TTACGAACTCCAGTTGTTCTCG | **Overlapping base** | 36 |  |
| LA-1 | **aaggagatatacaT**ATGTTCAACAGCGACAAC | **Overlapping base** | 32 |  |
| LA-2 | **Atgtatatctcctt**TTAACCAACTTTCACCGG | **Overlapping base** | 32 |  |

Note: *Underlines are restriction enzyme cutting site. Bolded underlines are overlapping bases from Overlap PCR.

**Supplementary Table 2.** Plasmids used in this study

| **Plasmids** | **Description** | **Sources** |
| --- | --- | --- |
| pET28a (+) | pBR322 ori, Promoter T7-lac, lacI, Kan^R^ | Novagen |
| pACYCDuet-1 | Double T7 promoters, p15A ori, Cm^R^ | Novagen |
| pETDuet-1 | Double T7 promoters, pBR322 ori, Amp^R^ | Novagen |
| pRSFDuet-1 | Double T7 promoters, RSF ori, Kan^R^ | Novagen |
| pET28a-7α | pET28a (+) carrying *7α-hsdh* | This study |
| pET28a-7β | pET28a (+) carrying *7β-hsdh* | This study |
| pET28a-ldh | pET28a (+) carrying *ldh* | This study |
| pET28a-gdh | pET28a (+) carrying *gdh* | This study |
| pACYCDuet-ldh-7α | pACYCDuet-1 carrying *ldh* and *7α-hsdh* | This study |
| pETDuet-ldh-7α | pETDuet-1 carrying *ldh* and *7α-hsdh* | This study |
| pRSFDuet-ldh-7α | pRSFDuet-1 carrying *ldh* and *7α-hsdh* | This study |
| pACYCDuet-ldh-7α | pACYCDuet-1 carrying *ldh* and *7α-hsdh* | This study |
| pETDuet-ldh-7α | pETDuet-1 carrying *ldh* and *7α-hsdh* | This study |
| pRSFDuet-ldh-7α | pRSFDuet-1 carrying *ldh* and *7α-hsdh* | This study |
| pRSFDuet-7β-7α | pRSFDuet-1 carrying *7β-hsdh* and *7α-hsdh* | This study |
| pACYCDuet-gdh-7β-ldh-7α | Each of the four genes in the pACYCDuet-1 vector has an independent T7 promoter | This study |
| pETDuet-gdh-7β-ldh-7α | Each of the four genes in the pETDuet-1 vector has an independent T7 promoter | This study |
| pRSFDuet-gdh-7β-ldh-7α | Each of the four genes in the pRSFDuet-1 vector has an independent T7 promoter | This study |
| pRSFDuet-gb-la | pRSFDuet-1 vector, *gdh*/7*β-hsdh* and *ldh*/*7α-hsdh* use a common T7 promoter, respectively | This study |
| pRSFDuet-gb-ldh-7α | pRSFDuet-1 vector, *gdh* and 7*β-hsdh* use a common T7 promoter, *ldh* and *7α-hsdh* has an independent T7 promoter | This study |
| pRSFDuet-gdh-7β-la | pRSFDuet-1 vector, *ldh* and 7*α-hsdh* use a common T7 promoter, *gdh* and *7β-hsdh* has an independent T7 promoter | This study |

**Supplementary Table 3.** Strains used in this study

| **Strains** | **Description** | **Resistance** | **Sources** |
| --- | --- | --- | --- |
| *E. coli* Top 10 | *F−mcrA Δ(mrr‐hsdRMS‐mcrBC) Φ80lacZΔM15 ΔlacX74 recA1 araD139 Δ(ara‐leu)7697 galU galK rpsL(Strr) endA1 nupG* | No | Laboratory |
| *E. coli* BL21 (DE3) | *F-dcm ompT hsdS (rB-mB-) gal λ(DE3)* | No | Laboratory |
| *E. coli* UCA01 | *E. coli* BL21(DE3) harboring pET28a-7α | Kan | This study |
| *E. coli* UCA02 | *E. coli* BL21(DE3) harboring pET28a-7β | Kan | This study |
| *E. coli* UCA03 | *E. coli* BL21(DE3) harboring pET28a-ldh | Kan | This study |
| *E. coli* UCA04 | *E. coli* BL21(DE3) harboring pET28a-gdh | Kan | This study |
| *E. coli* UCA05 | *E. coli* BL21(DE3) harboring pACYCDuet-ldh-7α | Cm | This study |
| *E. coli* UCA06 | *E. coli* BL21(DE3) harboring pETDuet-ldh-7α | Amp | This study |
| *E. coli* UCA07 | *E. coli* BL21(DE3) harboring pRSFDuet-ldh-7α | Kan | This study |
| *E. coli* UCA08 | *E. coli* BL21(DE3) harboring pACYADuet-gdh-7β | Cm | This study |
| *E. coli* UCA09 | *E. coli* BL21(DE3) harboring pETDuet-gdh-7β | Amp | This study |
| *E. coli* UCA10 | *E. coli* BL21(DE3) harboring pRSFDuet-gdh-7β | Kan | This study |
| *E. coli* UCA11 | *E. coli* BL21(DE3) harboring pRSFDuet-7β-7α | Kan | This study |
| *E. coli* UCA12 | *E. coli* BL21(DE3) harboring pACYADuet-ldh-7α and pRSFDuet-gdh-7β | Cm and Kan | This study |
| *E. coli* UCA13 | *E. coli* BL21(DE3) harboring pACYADuet-ldh-7α and pETDuet-gdh-7β | Cm and Amp | This study |
| *E. coli* UCA14 | *E. coli* BL21(DE3) harboring pRSFDuet-ldh-7α and pACYADuet-gdh-7β | Kan and Cm | This study |
| *E. coli* UCA15 | *E. coli* BL21(DE3) harboring pRSFDuet-ldh-7α and pETDuet-gdh-7β | Kan and Amp | This study |
| *E. coli* UCA16 | *E. coli* BL21(DE3) harboring pETDuet-ldh-7α and pACYADuet-gdh-7β | Amp and Cm | This study |
| *E. coli* UCA17 | *E. coli* BL21(DE3) harboring pETDuet-ldh-7α and pRSFDuet-gdh-7β | Amp and Kan | This study |
| *E. coli* UCA18 | *E. coli* BL21(DE3) harboring pACYADuet-gdh-7β-ldh-7α | Cm | This study |
| *E. coli* UCA19 | *E. coli* BL21(DE3) harboring pETDuet-gdh-7β-ldh-7α | Amp | This study |
| *E. coli* UCA20 | *E. coli* BL21(DE3) harboring pRSFDuet-gdh-7β-ldh-7α | Kan | This study |
| *E. coli* UCA21 | *E. coli* BL21(DE3) harboring pRSFDuet-gb-la | Kan | This study |
| *E. coli* UCA22 | *E. coli* BL21(DE3) harboring pRSFDuet-gb-ldh-7α | Kan | This study |
| *E. coli* UCA23 | *E. coli* BL21(DE3) harboring pRSFDuet-gdh-7β-la | Kan | This study |


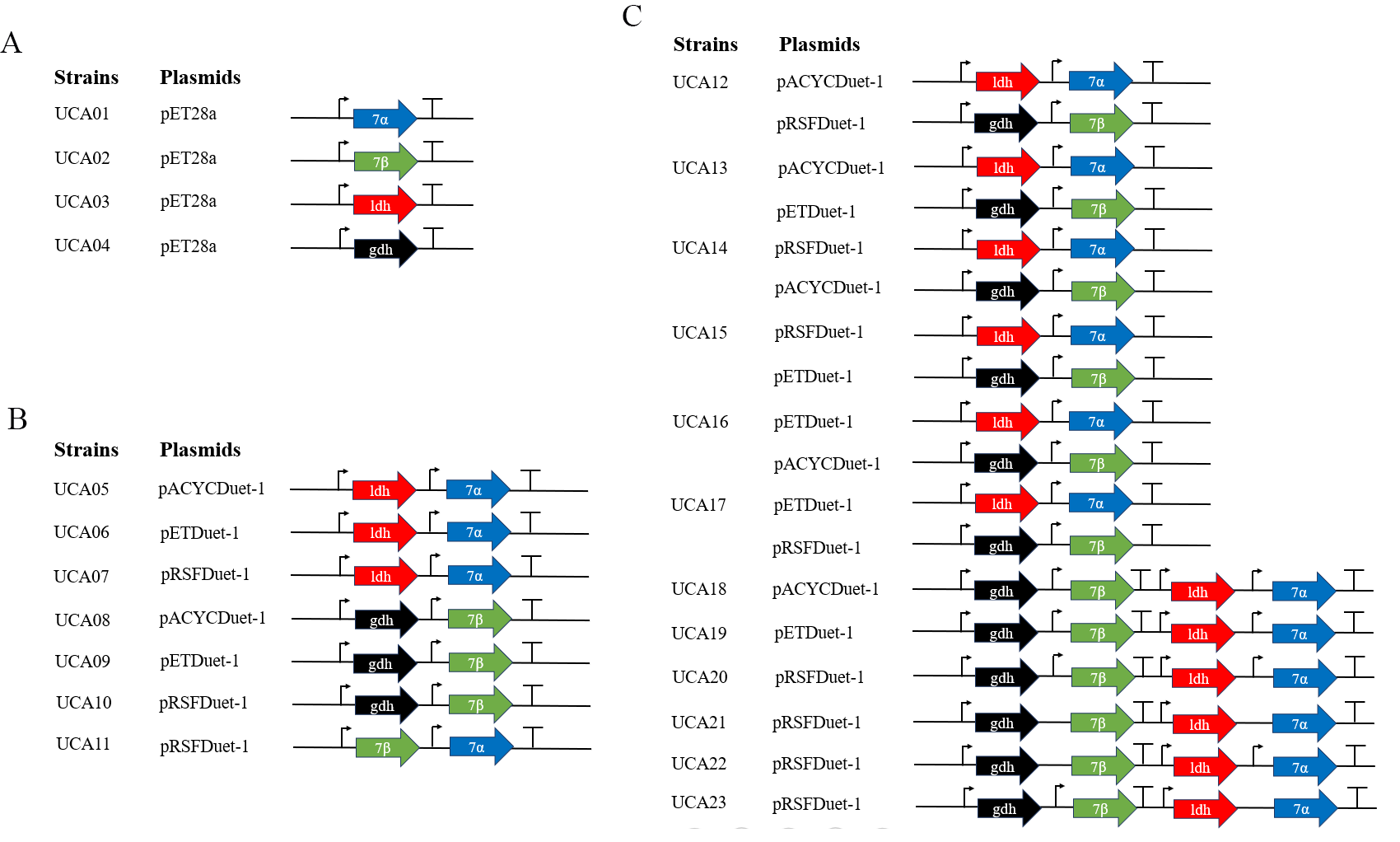


**Supplementary Figure 1.** Strains constructed in this study. (**A**)Construction of engineered strains expressing a single enzyme. (**B**)Construction of engineered strains coexpressing two enzymes. (**C**)Construction of engineered strains coexpressing four enzymes.


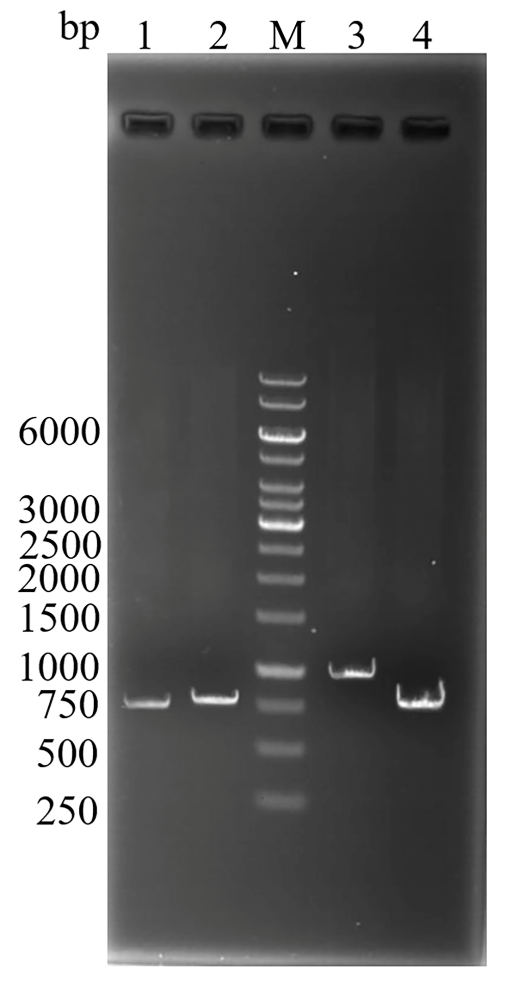


**Supplementary Figure 2.** Agarose gel electrophoresis of 7α-HSDH, 7β-HSDH, LDH and GDH gene from the strain UCA01 (lane 1), UCA02 (lane 2), UCA03 (lane 3), UCA04 (lane 4) amplified by PCR. Lane M: DNA ladder.


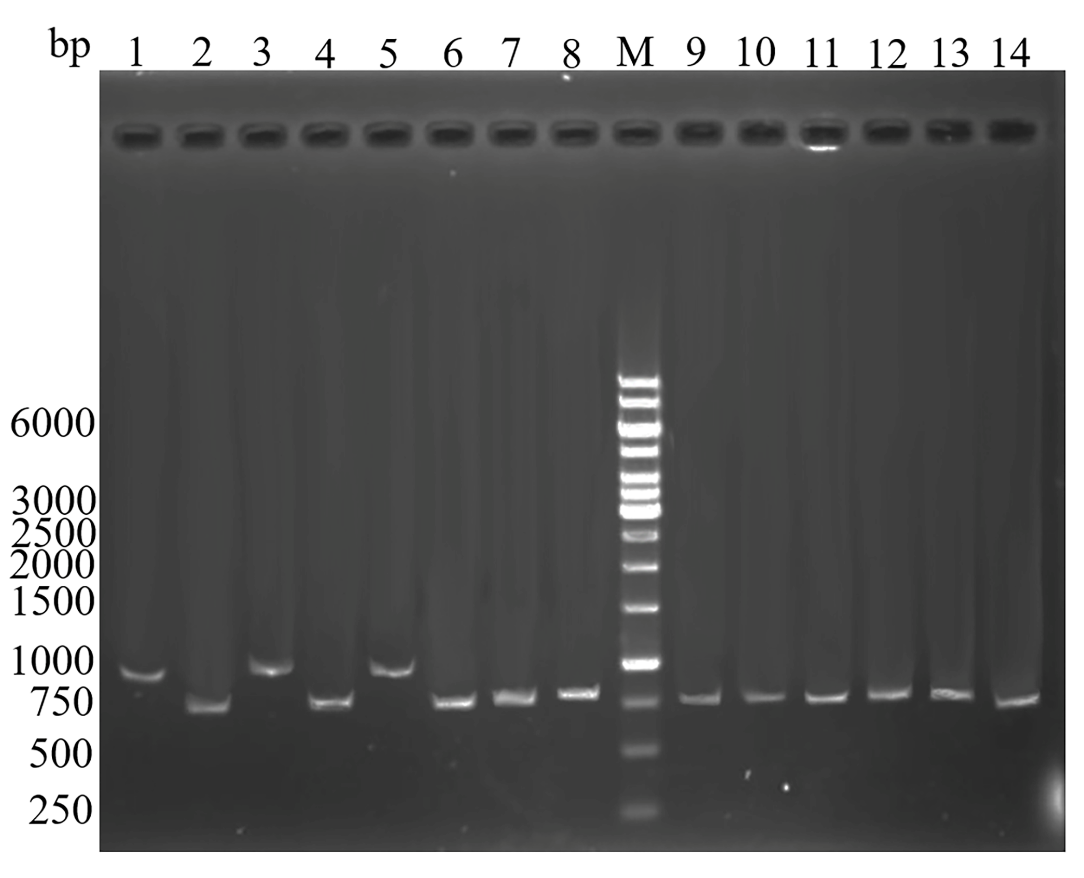


**Supplementary Figure 3.** Agarose gel electrophoresis of LDH/7α-HSDH, GDH/7β-HSDH and 7β-HSDH/7α-HSDH gene from the strain UCA05 (lane1-2), UCA06 (lane 3-4), UCA07 (lane 5-6), UCA 08(lane 7-8), UCA09 (lane 9-10), UCA10 (lane 11-12) and UCA11 (lane 13-14) amplified by PCR. Lane M: DNA ladder.


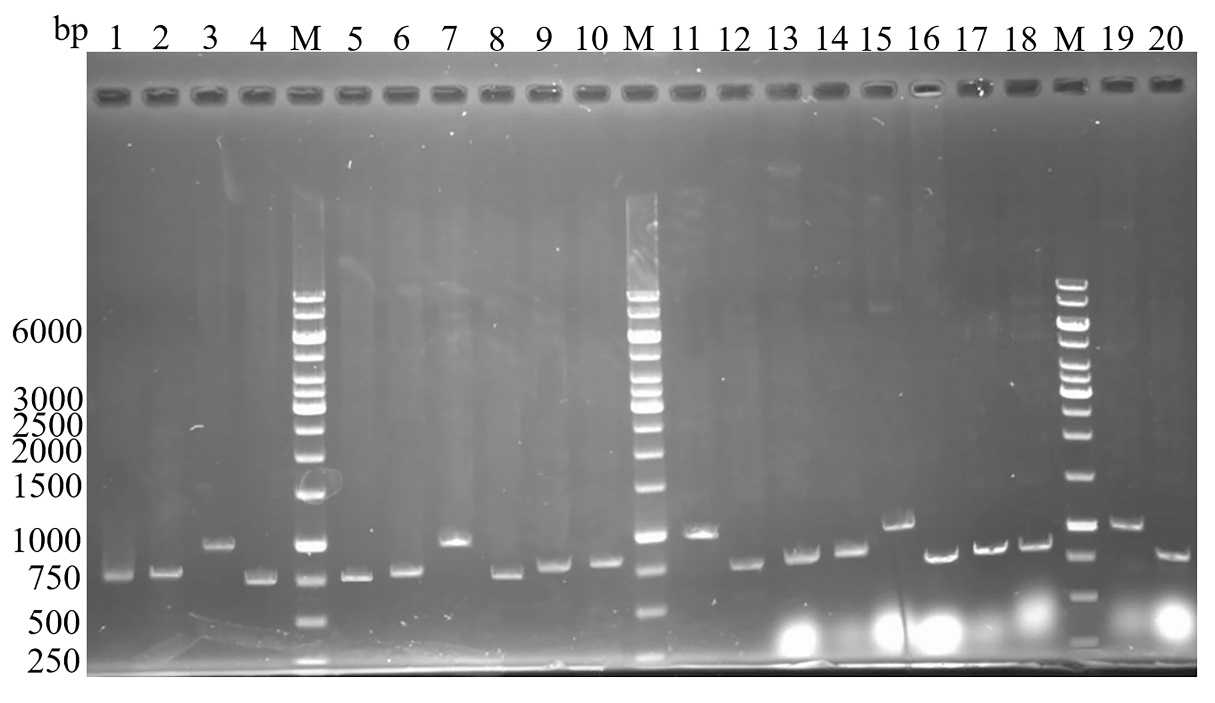


**Supplementary Figure 4.** Agarose gel electrophoresis of LDH, 7α-HSDH, GDH and 7β-HSD gene from the strain UCA18 (lane1-4), UCA19 (lane 5-8), UCA20 (lane 9-12), UCA21 (lane 13-16) and UCA22 (lane 17-20) amplified by PCR. Lane M: DNA ladder.

**
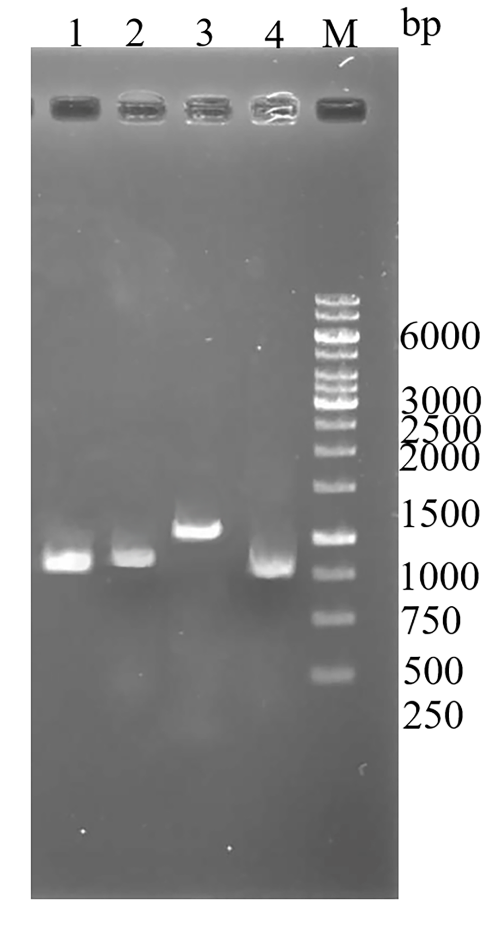
**

**Supplementary Figure 5.** Agarose gel electrophoresis of LDH, 7α-HSDH, GDH and 7β-HSDH gene from the strain UCA23 (lane 1-4) amplified by PCR. Lane M: DNA ladder.


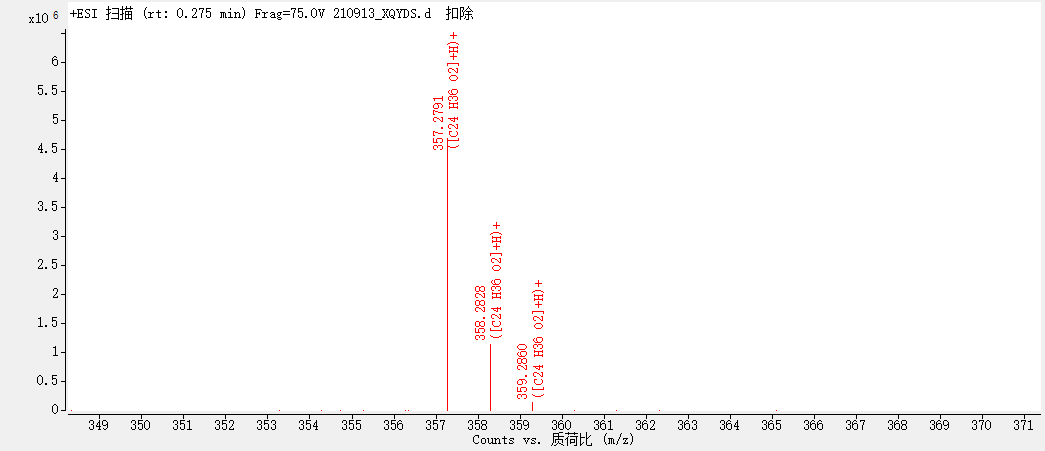


(A)The main peak (357.2791) is the plus H peak of UDCA stripped of 2 waters


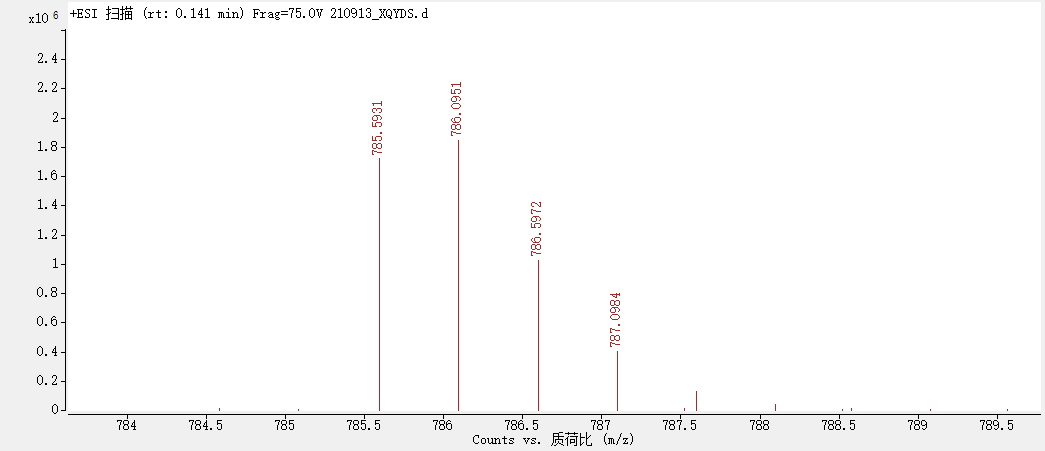


(B)The main peak (786.0951) is a 2-molecule UDCA+H peak

**Supplementary Figure 6.** Mass spectra of UDCA (C_24_H_40_O_4_, molecular weight: 392.58) structure identification.


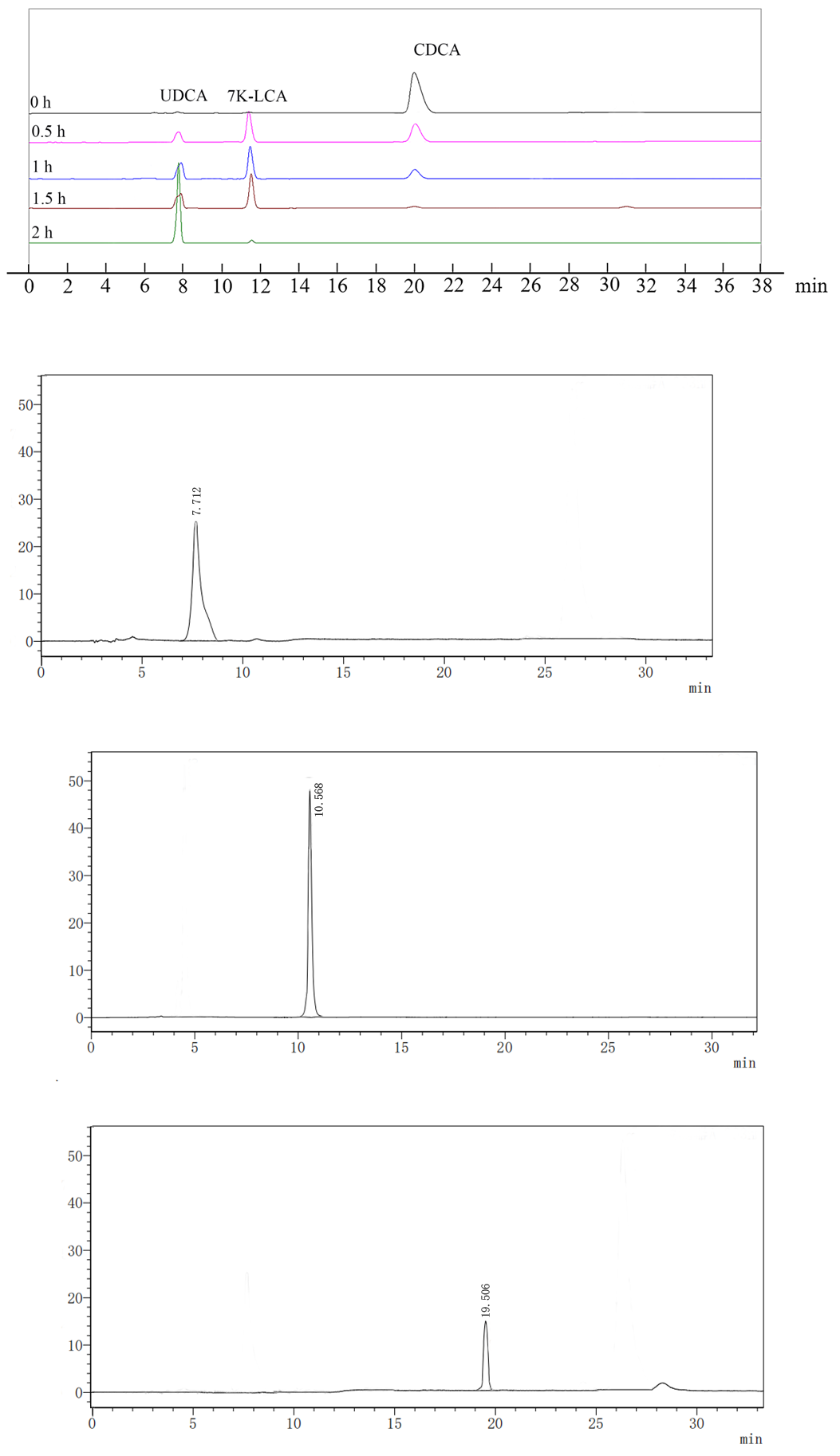


(A)HPLC chromatograms of standard UCDA


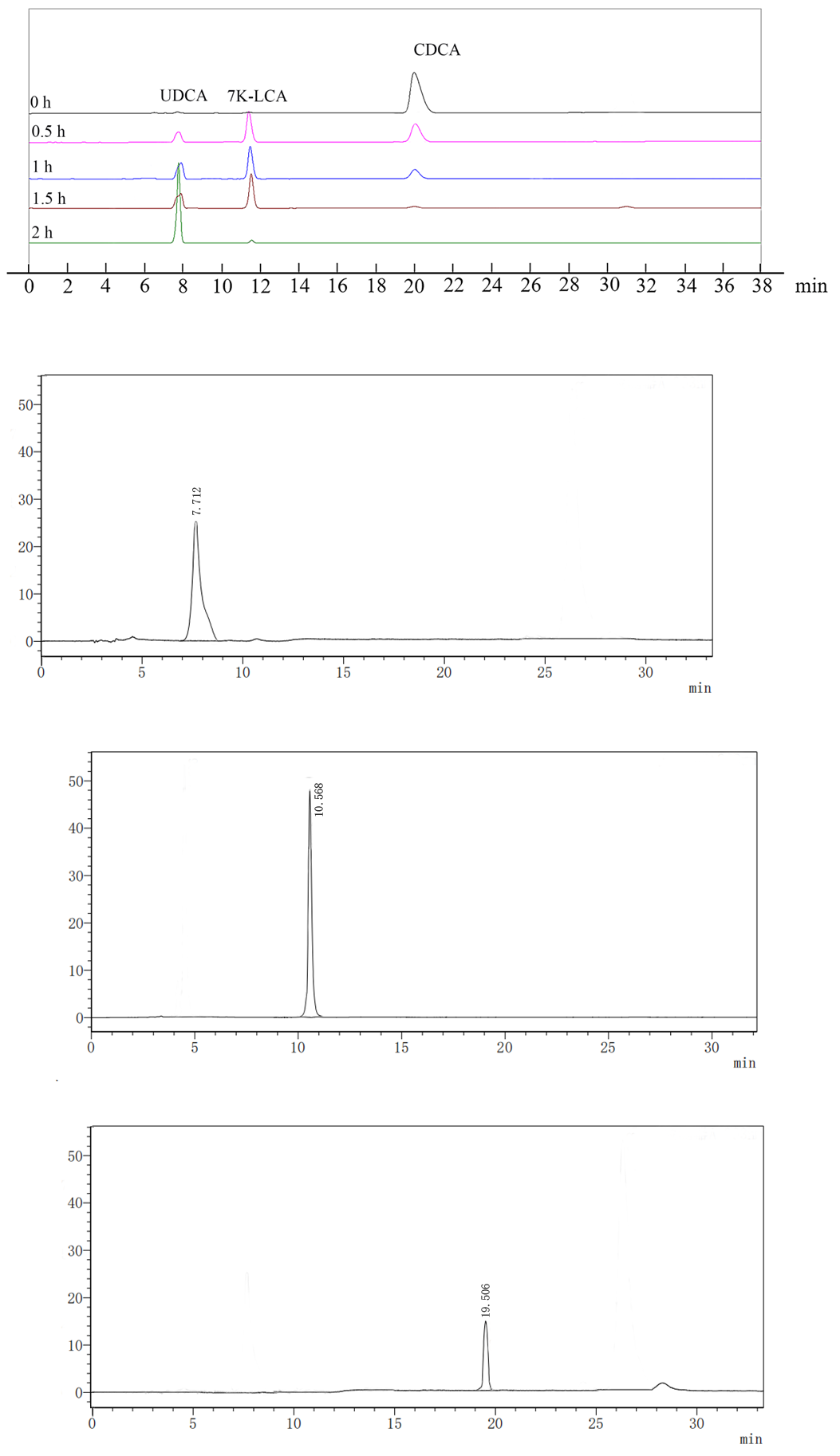


(B)HPLC chromatograms of standard 7K-LCA


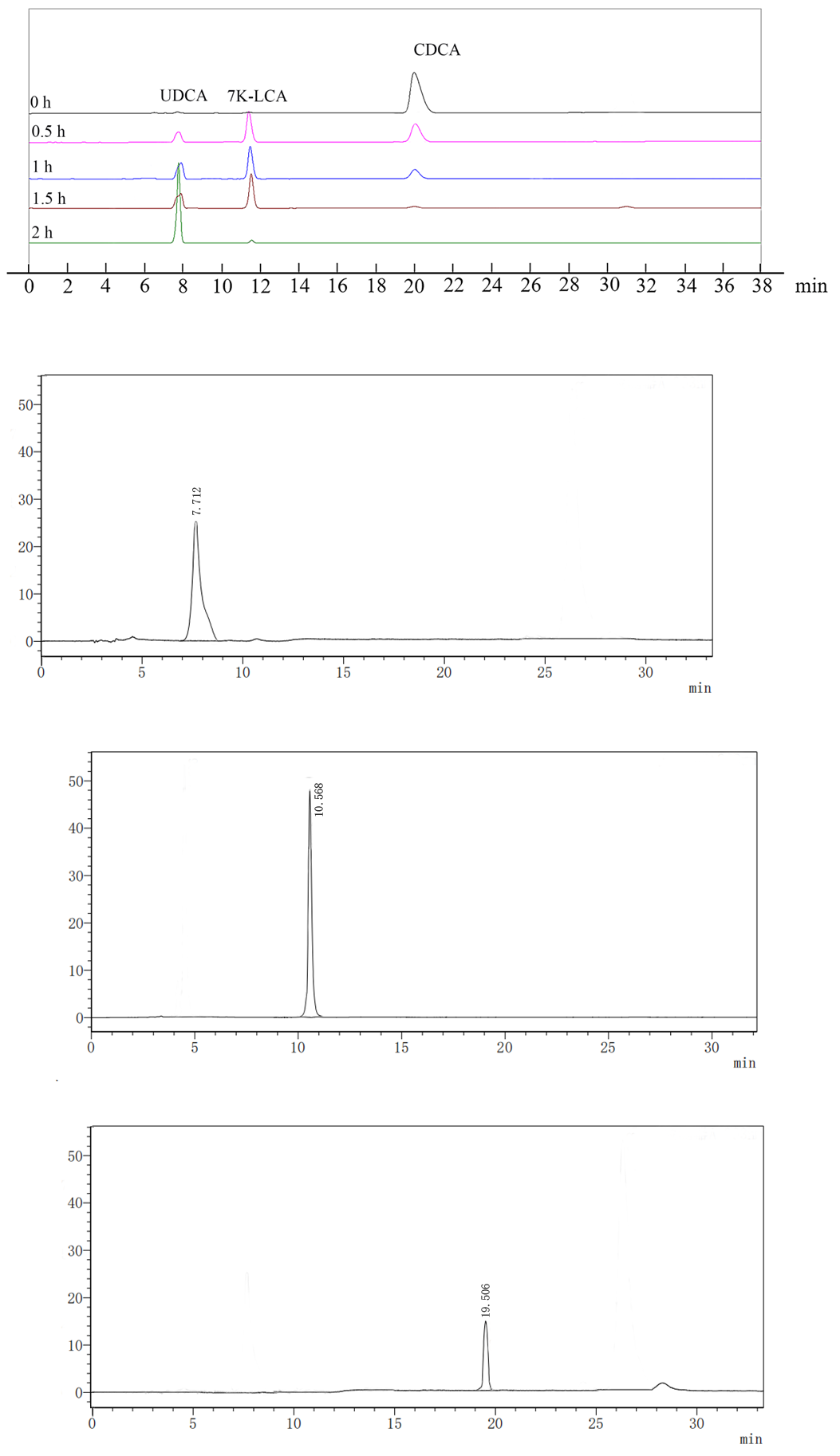


(C)HPLC chromatograms of standard CDCA

**Supplementary Figure 7.** HPLC chromatograms of standard UCDA, 7K-LCA, and CDCA
